# Powering the ABC multidrug exporter LmrA: How nucleotides embrace the ion-motive force

**DOI:** 10.1126/sciadv.aas9365

**Published:** 2018-09-19

**Authors:** Kelvin Agboh, Calvin H. F. Lau, Yvonne S. K. Khoo, Himansha Singh, Sagar Raturi, Asha V. Nair, Julie Howard, Marco Chiapello, Renata Feret, Michael J. Deery, Satoshi Murakami, Hendrik W. van Veen

**Affiliations:** 1 Department of Pharmacology, University of Cambridge, Tennis Court Road, Cambridge CB2 1PD, UK.; 2 Cambridge Centre for Proteomics, University of Cambridge, Cambridge CB2 1GA, UK.; 3 Department of Life Science and Technology, Tokyo Institute of Technology, Nagatsuta, Midori-ku, Yokohama 226-8501, Japan.

## Abstract

LmrA is a bacterial ATP-binding cassette (ABC) multidrug exporter that uses metabolic energy to transport ions, cytotoxic drugs, and lipids. Voltage clamping in a Port-a-Patch was used to monitor electrical currents associated with the transport of monovalent cationic HEPES^+^ by single-LmrA transporters and ensembles of transporters. In these experiments, one proton and one chloride ion are effluxed together with each HEPES^+^ ion out of the inner compartment, whereas two sodium ions are transported into this compartment. Consequently, the sodium-motive force (interior negative and low) can drive this electrogenic ion exchange mechanism in cells under physiological conditions. The same mechanism is also relevant for the efflux of monovalent cationic ethidium, a typical multidrug transporter substrate. Studies in the presence of Mg-ATP (adenosine 5′-triphosphate) show that ion-coupled HEPES^+^ transport is associated with ATP-bound LmrA, whereas ion-coupled ethidium transport requires ATP binding and hydrolysis. HEPES^+^ is highly soluble in a water-based environment, whereas ethidium has a strong preference for residence in the water-repelling plasma membrane. We conclude that the mechanism of the ABC transporter LmrA is fundamentally related to that of an ion antiporter that uses extra steps (ATP binding and hydrolysis) to retrieve and transport membrane-soluble substrates from the phospholipid bilayer.

## Teaser

Researchers study how different forms of metabolic energy are coupled to drug extrusion by an ATP-binding cassette transporter.

## INTRODUCTION

Adenosine 5′-triphosphate (ATP)–binding cassette (ABC) multidrug exporters are present in plasma membranes of all organisms (*1*). They can confer drug resistance on cells and affect drug pharmacokinetics and are intensely studied in clinical investigations (*2*). Previous studies on LmrA from *Lactococcus lactis* and its mammalian homolog, the multidrug resistance P-glycoprotein (ABCB1), highlight their ability to transport structurally diverse amphiphilic compounds from the plasma membrane where these compounds accumulate (*3*, *4*). The photoaffinity labeling of specific protein domains and regions in LmrA and other multidrug efflux pumps with transported drug analogs (*5*–*7*), the selective effects of site-directed mutagenesis on the polyspecificity for drugs (*8*), and the structural and biophysical observations on multidrug efflux pumps bound to ligands (*8*, *9*) demonstrate that the drug efflux reaction is based on direct protein-drug interactions. ABC transporters usually contain two membrane domains (MDs) that form the translocation pathway for transported substrates and two nucleotide-binding domains (NBDs) that bind and hydrolyze ATP. The functional LmrA transporter is a homodimer of two half-transporters, each containing an N-terminal MD fused to an NBD (*5*, *10*). The metabolic energy for substrate transport by ABC transporters is thought to be solely derived from ATP binding and hydrolysis, but observations on apparent proton-drug symport by LmrA (*11*) and proton-drug antiport by the lipid-A transporter MsbA from *Escherichia coli* (*12*) indicate that energy coupling to these ABC exporters is more complex. In addition, macroscopic ion currents have been detected in planar phospholipid bilayers for a truncated version of LmrA (termed LmrA-MD) containing the MD but lacking the NBD (*10*). It is pivotal for our understanding of how LmrA operates to examine the relationship between ion currents, proton-substrate cotransport, and ATP dependence in transport. Here, we applied electrophysiological and biochemical techniques to further study the mechanism of LmrA. As it is difficult to synchronize catalytic reactions in an ensemble of ABC transporters, our electrophysiological experiments with single-LmrA transporters in a phospholipid bilayer were of key importance for our ability to unravel the sequence of steps in energy coupling. We demonstrate, for the first time, sodium-motive force–dependent drug antiport in the ATP-bound state of LmrA and the ion stoichiometry of this reaction, and we assign functional roles to ion coupling and nucleotide utilization in the transport activity of this multidrug transporter.

## RESULTS

### Ion conductance by full-length LmrA

**Fig. 1:**
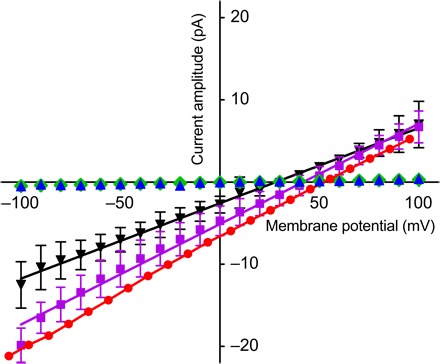
Current-voltage responses for different buffer compositions. LmrA-WT–containing phospholipid bilayers were subjected to a voltage step protocol with 10-mV steps for 1 s (ranging from −100 to +100 mV) from a holding potential of 0 mV. Macroscopic currents were recorded in symmetric buffer solutions containing 10 mM HEPES, 20 mM EGTA, and 10 mM NaCl (pH 7.2, NaOH) (blue triangle) or solutions containing asymmetric salt concentrations of 10 mM_in_/100 mM_out_ NaCl (red circle), LiCl (purple square), KCl (black triangle), or equimolar Na_2_SO_4_ (green diamond). Data points (mean ± SEM) are based on at least three independent measurements.

**Fig. 2:**
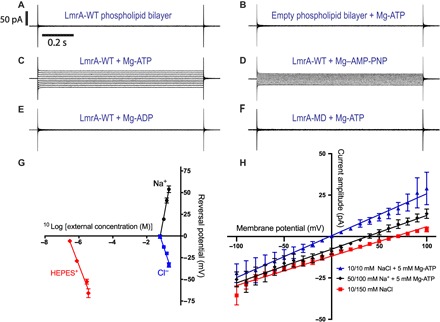
Ion conductance in response to nucleotide binding. (**A** to **F**) Macroscopic current responses to the imposition of the same voltage protocol as in Fig. 1, in symmetric 10 mM HEPES, 20 mM EGTA, and 10 mM NaCl solutions (pH 7.2, NaOH) in the absence or presence of 5 mM Mg-nucleotide in the external buffer. Scale bars in (A) are applicable to all current responses. (**G**) Determination of ion stoichiometry from *E*_rev_ measurements. *E*_rev_ was measured in the presence of 5 mM Mg-ATP as a function of the log_10_ of the external concentration of single buffer components: Na^+^ (black circle), Cl^−^ (blue square), or monovalent cationic HEPES^+^ (red diamond). Data points (±SEM) show the mean *E*_rev_ for at least three independent measurements. The internal concentration of Na^+^ and Cl^−^ was maintained at 60 mM, and the external concentration was raised from 60 to 100, 150, or 200 mM, with HEPES maintained at 10 mM in internal and external solutions. To measure the effect of HEPES (pH 6.5), the internal solution contained 60 mM NaCl, 20 mM EGTA, and 10 mM HEPES. The external HEPES concentration was raised from 10 to 25, 100, or 125 mM, yielding HEPES^+^ concentrations of 0.29, 0.73, 2.9, and 3.6 μM. The ion stoichiometry was determined from the slope of the linear fit using the Nernst equilibrium equation (data analysis S1). The slope values for Na^+^, Cl^−^, and HEPES^+^ were 104.6 ± 5.3 mV, −59.1 ± 7.8 mV, and −51.1 ± 4.9 mV, respectively (*n* = 3). (**H**) Current-voltage relation (*I*-*V*) response graph for LmrA-WT using symmetric solutions as listed under (A) with 5 mM Mg-ATP in the external buffer (blue trace), or asymmetric solutions containing [Na^+^]_in_/[Na^+^]_out_ = 50 mM/100 mM and [Cl^−^]_in_/[Cl^−^]_out_ = 50 mM/50 mM in the presence of the ATP (black trace), or containing [NaCl]_in_/[NaCl]_out_ = 10 mM/150 mM without nucleotide (red trace). The *E*_rev_ for the blue, black, and red traces was −2.0 ± 1.2 mV, 37.6 ± 1.5 mV, and 66.7 ± 6.1 mV (*n* = 3), respectively.

In previous work, purified LmrA-MD inserted in a phospholipid bilayer showed ion conductance when probed with the tip-dip technique in an electrophysiology rig (*10*). These measurements showed that Cl^−^ and Na^+^ significantly contribute to the ion current observed. The work described here started with control experiments in which the relevance of these observations was tested for purified full-length LmrA (LmrA-WT) in a phospholipid bilayer in a miniaturized Nanion Port-a-Patch setup (Fig. 1 and fig. S1). The current responses to voltage steps (membrane potential) demonstrate that the mere presence of LmrA-WT proteins in bilayers with symmetric external and internal solutions each containing either 10 or 100 mM NaCl did not cause a significant change in ion conductance from that of empty bilayers. However, we observed two conditions where significant current responses were obtained at a high frequency (82 of a total of 183 experiments). First, the imposition of the membrane potential and an inwardly directed NaCl gradient ([NaCl]_in_/[NaCl]_out_ = 10 mM/100 mM) produced noticeable ion currents for LmrA-WT–containing bilayers (Fig. 1). Consistent with the earlier observations of LmrA-MD, Na^+^ could effectively be replaced by Li^+^ but only weakly by K^+^. With the use of SO_4_^2−^ instead of Cl^−^ (with Na^+^ maintained), there was no current response at all, suggesting that Cl^−^ is essential in the ion conductance. Second, at symmetric NaCl solutions ([NaCl]_in_/[NaCl]_out_ = 10 mM/10 mM) in the presence of membrane potential as a driving force for ion movement, the application of 5 mM Mg-ATP in the external solution induced a current response for LmrA-WT–containing phospholipid bilayers (Fig. 2, A to C). This response was also obtained with 5 mM of the slowly hydrolyzable ATP analog Mg–AMP-PNP (adenylyl-imidodiphosphate) (Fig. 2D), pointing to a role of ATP binding rather than ATP hydrolysis in the initiation of the current. No current response was observed when LmrA-MD (lacking the NBD) was used instead of LmrA-WT, or when Mg-ADP (adenosine 5′-diphosphate) replaced the Mg-ATP (Fig. 2, E and F). Thus, the imposition of membrane potential with ATP binding at the NBDs in the transporter (Fig. 2) or the simultaneous imposition of membrane potential and chemical ion gradients (Fig. 1) can each initiate LmrA-WT–associated ion conductance. Previously, nanoelectrospray mass spectrometry analysis confirmed that LmrA-WT forms a homodimer without additional binding partners (*10*). Here, liquid chromatography–tandem mass spectrometry (LC-MS/MS) analyses of our LmrA-WT preparations further demonstrate the absence of contaminating membrane proteins (fig. S2 and table S1). The current responses are therefore directly linked to native LmrA-WT protein rather than to an exogenous ion translocator that is regulated by LmrA.

### Determination of ion stoichiometry

To investigate the ion stoichiometry and relative directions of ATP-induced ion movement for LmrA-WT, the membrane potential where the net flux of ions is zero and the ionic current is absent, also referred to as the reversal potential (*E*_rev_), was measured as a function of the external concentration of single ions (Fig. 2G and data analysis S1, A to D). An increase in the external Na^+^ concentration resulted in a positive shift of *E*_rev_, whereas an increase in the external Cl^−^ concentration resulted in a negative shift (Fig. 2G). Measurements of the concentration dependence of these shifts demonstrate the translocation of 2Na^+^ ions in antiport with 1Cl^−^ (data analysis S1, A and B). With the previously established symport of Cl^−^ and H^+^ at a 1:1 ratio (*10*), analysis of the charge balance in the ion exchange reaction and subsequent *E*_rev_ measurements indicate the translocation of one monovalent cationic HEPES^+^ (protonated on the piperazin and sulfonate moieties) per transport cycle (data analysis S1C). These experiments point to 2Na^+^/(1H^+^-1HEPES^+^-1Cl^−^)^+^ exchange with a net transmembrane movement of one positive charge per cycle (data analysis S1D). Ion exchange was further examined in *E*_rev_ measurements for Mg-ATP binding–induced currents with symmetric or asymmetric buffer solutions in which the Na^+^ and Cl^−^ concentrations were changed simultaneously. Under these conditions, the observed *E*_rev_ values were very close to the calculated *E*_rev_ values for 2Na^+^/(1H^+^-1HEPES^+^-1Cl^−^)^+^ exchange (Fig. 2H and data analysis S1E) but did not match predictions for ion transport with other stoichiometries. Thus, ion conductance by LmrA can be described using thermodynamic driving forces for secondary-active membrane transporters.

### Ion currents associated with single-LmrA transporters

**Fig. 3:**
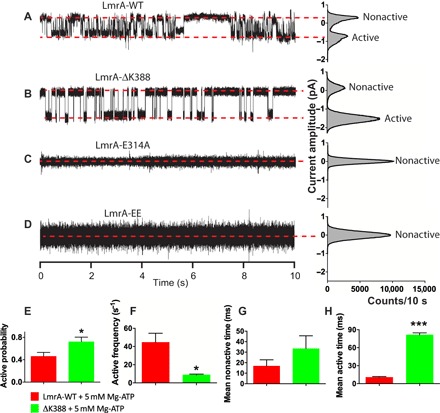
Ion conductance is initiated by Mg-ATP binding and terminated by ATP hydrolysis. (**A** to **D**) Single-transporter current responses to the imposition of a membrane potential of −50 mV with symmetric solutions containing 10 mM NaCl, 50 mM KCl, 10 mM HEPES, and 20 mM EGTA (pH 7.2, NaOH) in the presence of Mg-ATP in the external buffer. Red dashed lines refer to nonactive and active states (usually referred to as closed and open states in channel studies). All-point amplitude histograms presented on the right of every recording were obtained using an equivalent current range for all cases (bin count = 400). Mg-ATP fails to induce single-transporter conductance for transport-inactive mutants LmrA-E314E and LmrA-EE, which were inserted in the bilayer at a similar protein concentration as LmrA-WT. (**E** to **H**) Analyses of nonactive and active time distributions from which relevant parameters are derived [*P < 0.05, ***P < 0.001, one-way analysis of variance (ANOVA)]. Inhibition of ATP hydrolysis prolongs the time period during which ion exchange is observed.

Since ATP addition to LmrA-containing lipid bilayers can elicit ion flux, we investigated the possibility of recording single-LmrA currents (Fig. 3, A to D). A lipid bilayer was formed after the reconstitution of 1 to 5 pmol of LmrA into giant unilamellar phospholipid vesicles. The resultant bilayer was clamped at a membrane voltage of −50 mV. Similar to the macroscopic data, the application of 5 mM Mg-ATP elicited current responses with inwardly directed, downward deflections with respect to the baseline (Fig. 3A). We focused on single transporter responses by excluding sections where signal stacking was observed (equivalent to channel stacking where more than one channel is open at the same time). Such current responses were obtained in 34% of our attempts (61 of a total of 179 experiments), which is excellent given the significant dilution of LmrA protein in these experiments. In control studies with equal amounts of protein, the E314A and EE mutants that are inactive in proton-Cl^−^ symport (*10*) never produced observable currents in the presence of 5 mM Mg-ATP (Fig. 3, C and D). To test the role of ATP binding versus ATP hydrolysis in ion transport, experiments were performed using the adenosine triphosphatase (ATPase)–impaired ΔK388 mutant (Fig. 3B) (*13*). The mutant exhibits only 4% of the ATPase activity of LmrA-WT because of the deletion of the Walker A lysine (ΔK388) in the NBD (*14*). In LC-MS/MS analyses, our samples for this mutant protein are devoid of contaminating membrane proteins (fig. S2 and table S2). The addition of 5 mM Mg-ATP to lipid bilayers containing the LmrA-ΔK388 mutant elicited single-transporter current responses with a significantly decreased number of transitions per unit time (active frequency) and increased mean time during which LmrA is transport-active (mean active time) (Fig. 3, E to H). The results indicate that the initiation of ion exchange in LmrA requires ATP binding, whereas termination of ion exchange requires ATP hydrolysis; this latter step is delayed for LmrA-ΔK388 (Fig. 3H). This mechanism of nucleotide-dependent regulation of the ABC transporter LmrA shows analogy to the nucleotide-dependent gating of the mammalian cystic fibrosis transmembrane conductance regulator Cl^−^ channel, which represents another major class of membrane proteins in the ABC superfamily.

### Measurements of drug-ion exchange

**Fig. 4:**
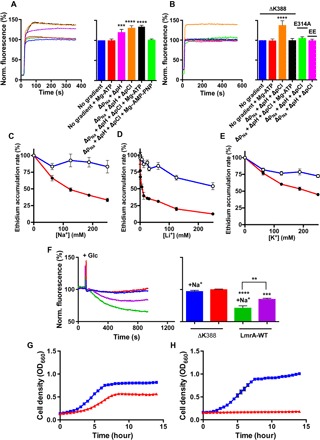
Ion exchange is linked to drug transport. (**A** and **B**) Transport of ethidium (2 μM) in DNA-loaded proteoliposomes containing LmrA-WT (A) or ATPase-deficient LmrA-ΔK388 or proton transport–deficient LmrA-E314A (B) in an inside-out fashion, in the absence or presence of nucleotide and/or the stepwise imposition of a sodium-motive force Δp_Na_ (interior positive and [Na^+^]_in_/([Na^+^]_out_ = 100 mM/1 mM), chemical proton gradient ΔpH (pH_in_ 8.0/pH_out_ 6.8), and chemical chloride gradient ΔpCl ([Cl^−^]_in_/([Cl^−^]_out_ = 50 mM/150 mM). Line colors are the same as bar colors and refer to the same experimental conditions. (**C** to **E**) Effect of salts on the ethidium accumulation rate in cells with (black circles) and without LmrA (white circles) as a percentage of the rate in the absence of salts. (**F**) Effect of 100 mM NaCl in the external buffer on ethidium efflux from cells preloaded with 2 μM ethidium. Efflux was initiated by the addition of 25 mM glucose (+Glc) as a source of metabolic energy. (**G** and **H**) Growth of *L. lactis* expressing LmrA-WT (blue trace) or LmrA-ΔK388 (red trace) in the presence of 4 μM ethidium in standard M17 medium (pH 7.0) (G) or M17 medium containing 16.7 mM NaOH (pH 7.5) plus 25 mM NaCl (H). The error bars for some of the data points are too small to be displayed and are hidden behind the data point symbols. Data represent observations in three or more independent experiments with independently prepared batches of proteoliposomes and cells. Values in histograms show significance of fluorescence levels at steady state and are expressed as means ± SEM (***P < 0.001, ****P < 0.0001, one-way ANOVA).

Given the speciation of HEPES buffer at the prevailing pH of 6.5 (data analysis S1C), LmrA-WT transports HEPES^+^ in a similar micromolar concentration range as typical multidrug transporter substrates such as monovalent cationic ethidium (*15*). As we previously obtained evidence for LmrA-mediated proton-ethidium symport (*11*), we examined 2Na^+^/(1H^+^-1ethidium^+^-1Cl^−^)^+^ exchange using proteoliposomes in which purified LmrA was inserted in an inside-out fashion (Fig. 4) (*11*). Ethidium uptake into these proteoliposomes is associated with a fluorescence intensity enhancement due to ethidium binding to DNA in the lumen. The stepwise imposition of an outwardly directed sodium-motive force (Δp_Na_, interior positive and high) and inwardly directed chemical H^+^ and Cl^−^ gradients (ΔpH and ΔpCl, interior alkaline and low, respectively) strongly stimulated the accumulation of ethidium (Fig. 4A). No ethidium transport was observed for the E314A substitution mutant of LmrA, which affects the glutamate side chain that is essential for proton-coupled drug transport (*11*, *15*). Ethidium transport was also lost for the K32E R34E (EE) double mutant, in which the replacement of basic residues in the N-terminal elbow helix by an acidic side chain leads to inactivation of LmrA (Fig. 4B) (*10*).

We examined the role of the ATPase activity in the LmrA-mediated ethidium ion–exchange reaction. Although the ion-coupled ethidium transport activity was not altered following the inclusion of Mg-ATP in the assay, the presence of the nonhydrolyzable nucleotide analog AMP-PNP strongly inhibited ethidium transport due to nucleotide trapping (Fig. 4A). This type of inhibition was also obtained following ATP binding to the ATPase-deficient LmrA-ΔK388 mutant (Fig. 4B). Thus, ethidium transport is ATP-binding and hydrolysis-dependent when the nucleotide is present but is dependent on electrochemical ion gradients in the absence of nucleotide.

LmrA-mediated ethidium ion exchange was also tested in *L. lactis* cells. The exposure of LmrA-WT–expressing cells to Na^+^ strongly stimulated the efflux of ethidium (Fig. 4, C and F). Consistent with the electrophysiological data in Fig. 1, this effect of Na^+^ was also observed with Li^+^ and, to a lesser extent, with K^+^ (Fig. 4, D and E). In accordance to the observations in proteoliposomes (Fig. 4, A and B), no ethidium efflux was observed in cells for the LmrA-ΔK388 mutant (Fig. 4F). Finally, LmrA-dependent ethidium resistance in lactococcal cells was strongly enhanced in M17 growth medium (pH 7.5 rather than the standard pH 6.9), which was supplemented with 41.7 mM Na^+^. Through the enhancement of the sodium-motive force (exterior positive and high Na^+^), this medium promotes Na^+^ uptake by LmrA. In addition, by raising pH_out_ to a value closer to pH_in_, this medium reduces the transmembrane proton gradient that in physiological settings would oppose H^+^ efflux (Fig. 4, G and H). Hence, 2Na^+^/(1H^+^-1ethidium^+^-1Cl^−^)^+^ exchange is directly relevant for LmrA’s ethidium transport activity in proteoliposomes and cells and its ability to confer ethidium resistance on cells.

### Ion binding sites in LmrA

In previous studies, we identified one of the ion binding residues, E314 in TM6 (transmembrane helix 6) in each half-transporter as being important for proton and ethidium transport (*10*, *11*, *15*). In a structure model of LmrA representing the ATP-bound, outward-facing state (*16*), E314 is located in the interior cavity at the dimer interface in close proximity to an amide-containing face (including Q141, N144, and N148) in TM3. The bottom of the interior cavity near the membrane-cytoplasm interface contains four amide side chains, N137 (intracellular extension of TM3) and Q211 (intracellular extension of TM4) in each half-transporter (fig. S3), the arrangement of which resembles the arrangement of key amide residues in crystallized proteins with Cl^−^ and Na^+^ binding sites that lack an ionized side chain of opposite charge in the coordination sphere (fig. S4). Specifically, a systematic search of high-resolution structures in the Protein Data Bank (see Materials and Methods) shows multiple examples of Na^+^ and Cl^−^ binding sites in which the ion is coordinated by three, four, or five Asn or Gln residues. For Na^+^, backbone carbonyl contacts or water molecules complete the coordination sphere (fig. S4A). For Cl^−^, the binding site is often completed with hydrophobic side chains, as one might expect for a polarizable ion (fig. S4B). Consistent with the notion that ions might bind at the interface between the two LmrA half-transporters, most of the identified Na^+^ and Cl^−^ binding sites featuring this chemical makeup are observed in oligomeric proteins in which they bridge an interaction between protomers. Hence, ATP-bound LmrA contains important candidate locations for H^+^, Na^+^, and Cl^−^ binding.

**Fig. 5:**
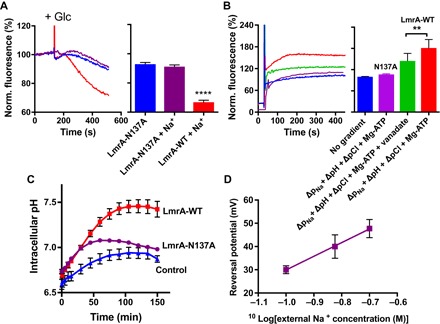
N137A mutation impairs drug and ion transport by LmrA. (**A**) Ethidium efflux from preloaded cells containing LmrA-N137A was performed as described in Fig. 4F and was initiated by the addition of 25 mM glucose (+Glc) as a source of metabolic energy. Transport in the absence or presence of 50 mM Na_2_SO_4_ is compared with the Na^+^-stimulated activity for LmrA-WT. Line colors are the same as bar colors and refer to the same experimental conditions. (**B**) Ethidium transport in proteoliposomes was measured with artificial imposition of the same ion gradients as described in Fig. 4 (A and B). Where indicated, LmrA-WT activity was inhibited by inclusion of 1 mM Na-vanadate in the assay buffer. (**C**) H^+^ efflux was measured in cells loaded with pH probe 5(6)-carboxyfluorescein diacetate succinimidyl ester (CFDASE) to monitor the intracellular pH in the absence or presence of 125 mM Na_2_SO_4_ in the external buffer (based on up to nine independent measurements). Metabolic energy was generated in the cells by the addition of glucose at *t* = 0 min. (**D**) *E*_rev_ was measured as described in Fig. 2G as a function of the log_10_ of the external Na^+^ concentration. The slope value was 58.8 ± 1.6 mV. Values in histograms show significance of fluorescence end levels and are expressed as means ± SEM (**P < 0.01, ****P < 0.0001, one-way ANOVA).

To analyze the importance of N137A in drug and ion transport by LmrA, the LmrA-N137A mutant protein was expressed in the plasma membrane of lactococcal cells at a similar level as LmrA-WT (fig. S5). In ethidium transport experiments in cells (Fig. 5A), the efflux activity of the N137A mutant was significantly reduced compared to LmrA-WT and not stimulated in the presence of 100 mM Na^+^. The reduced ethidium transport activity of the mutant was also observed in proteoliposomes in which the Δp_Na_ (interior positive and high), ΔpH (interior low), and ΔpCl (interior low) were imposed simultaneously (Fig. 5B). In contrast, LmtA-WT showed a robust, vanadate-inhibited activity in these proteoliposomes that was well above the empty-liposome control. Furthermore, similar to earlier observations for LmrA-E314A (*10*), the N137A mutation inhibited LmrA-dependent proton efflux in intact cells, resulting in a diminished alkalinization of the cytosol when the cells are exposed to Na^+^ (Fig. 5C). Finally, electrophysiological measurements of the *E*_rev_ for LmrA-N137A as a function of the external Na^+^ concentration show a significant twofold reduction in the Δ*E*_rev_/Δlog[Na^+^]_out_ slope value for the mutant compared to LmrA-WT (58.8 ± 1.6 mV versus 104.6 ± 5.3 mV; Fig. 2D), pointing to a reduced dependency of the *E*_rev_ on the chemical Na^+^ gradient and a change in the ion stoichiometry for the mutant. Ion conduction by LmrA-N137A is no longer associated with the Na^+^-stimulated ethidium transport of LmrA-WT.

## DISCUSSION

Previous observations on LmrA-MD–mediated ethidium-proton symport led to the conclusion that the MD dimer of the ABC exporter LmrA is functional as a secondary-active multidrug translocator (*11*, *15*). Here, the ion-coupled reaction underlying these observations and its ion stoichiometry were studied for the first time in detail for full-length LmrA using electrophysiological techniques. Surprisingly, LmrA can mediate apparent 2Na^+^/(1H^+^-1HEPES^+^-1Cl^−^)^+^ exchange, driven by the sodium-motive force under physiological conditions in which HEPES^+^ acts as an organic “drug-like” substrate. Early studies reported the interaction of mammalian ABCB1 with HEPES, but the mechanism of this interaction was not resolved (*17*). As the chemical gradients for Na^+^, Cl^−^, and H^+^ that drive HEPES^+^ transport also drive ethidium transport in proteoliposomes, the proposed mechanism is directly relevant for LmrA-mediated ethidium transport in cells (Fig. 4). 2Na^+^/(1H^+^-1ethidium^+^-1Cl^−^)^+^ exchange is consistent with earlier observations on LmrA-WT of apparent proton-ethidium symport and proton-chloride symport in proteoliposomes (data analysis S2) (*10*, *11*, *15*) and proton and chloride efflux from lactococcal cells (*10*). Given the equilibrium drug binding studies on LmrA indicating the binding of two vinblastine molecules per LmrA dimer (*5*), the proton in the ion exchange reaction might be effluxed instead of a second ethidium^+^ or HEPES^+^ molecule. In this model, substrates and protons would compete for binding to LmrA by direct or indirect mechanisms, as described for secondary-active multidrug transporters.

Although HEPES^+^ and ethidium share a common mechanism of transport, differences were observed in the regulation by nucleotide. In the presence of a membrane potential as a driving force for ion translocation (Figs. 2 and 3), ATP binding initiates the ion-coupled HEPES^+^-transport reaction, whereas ATP hydrolysis terminates the reaction. Repeats of these experiments with the ATPase-deficient ΔK388 mutant demonstrate the positive correlation between prolonged ATP binding and the increased lifetime of the ion exchange state (Fig. 3). Furthermore, the current response is also initiated upon binding of the slowly hydrolyzable nucleotide analog AMP-PNP (Fig. 2D). These results suggest that HEPES^+^ transport is associated with an ATP-bound state, as shown in current structures of outward-facing MsbA (*18*), Sav1866 (*19*), and PglK (*20*), as well as outward-occluded McjD (*21*) in which the NBDs are dimerized because of ATP binding at the dimer interface. In contrast to these observations for HEPES^+^ transport, ion-coupled ethidium transport is inactivated in response to AMP-PNP binding (Fig. 4A) or ADP·vanadate binding (Fig. 5B) to LmrA-WT or ATP binding to LmrA-ΔK388 (Fig. 4, B and F). Instead, ATP hydrolysis is compulsory for the ethidium transport activity, demonstrating a requirement for the ATP binding–induced NBD dimerization followed by ATP hydrolysis–dependent disruption of the NBD dimer interface. The disengagement of NBDs is observed in structures of inward-facing, nucleotide-free MsbA (*18*) and Atm1 (*22*), ADP-bound, outward-occluded PglK (*20*), and ADPβS-bound MacB (*23*). NBD association and dissociation have also been observed for LmrA in distance measurements by pulsed electron-electron double resonance (PELDOR/DEER) spectroscopy (*24*). Together with cysteine cross-linking studies (*25*), these distance measurements suggest a high similarity between prehydrolysis, transition, and posthydrolysis catalytic intermediate states of LmrA, indicating that ATP binding triggers one major conformational change in LmrA, while smaller rearrangements are required to complete the transport and hydrolysis cycles. The observed stoichiometric coupling between ion fluxes in LmrA is therefore most likely based on cooperative ion binding events and rapid movements of individual amino acid side chains rather than the movements of entire protein domains. Previous studies have raised the awareness that fast-acting membrane transporters can share functional and mechanistic features with ion channels. Our data suggest that similar considerations are true for LmrA.

One of the remarkable differences between HEPES^+^ and ethidium is their affinity for residence in a lipophilic environment. With partition coefficients in an octanol-water solvent system of 1:1 × 10^4^ for HEPES^+^ and 1.6 × 10^4^:1 for ethidium (*26*), the ability of these compounds to accumulate in the phospholipid bilayer is very low for HEPES^+^ and very high for ethidium. By analogy to proton-motive force–dependent Resistance-Nodulation-Cell Division (RND)–type multidrug transporters, in which the drug-binding domains contain distinct drug entry pathways that enable access of substrates from the aqueous periplasm and the lipophilic outer leaflet of the plasma membrane (*27*–*29*), we propose that LmrA has an aqueous pathway for substrate transport from the cytosol and a hydrophobic pathway for substrate transport from the membrane. The measurements on the nucleotide dependence of HEPES^+^ transport indicate that the aqueous pathway is associated with the ATP-bound state of LmrA (Figs. 2 and 3). However, the observations on ion-coupled HEPES^+^ and ethidium transport in the absence of ATP (Figs. 1, 2H, and 4, A and B) suggest that the aqueous pathway is also active in apo-LmrA. PELDOR/DEER measurements for LmrA have shown that the ATP-bound state can be sampled in the absence of nucleotide because of the significant orientational distribution of NBD and MDs in LmrA’s apo state (*24*). In the hydrophobic pathway, steps in the ATP hydrolysis cycle most likely regulate the extraction of lipid-soluble substrates from the inner leaflet of the phospholipid bilayer and/or the delivery of these substrates into the outer leaflet of the membrane. The data on ion-coupled ethidium transport in the absence of ATP suggest that the hydrophobic pathway converges with the aqueous pathway in the translocation route and that small molecules such as ethidium can diffuse through the hydrophobic pathway when the regulation by ATP is not imposed. Consistent with the measurements of ethidium transport in proteoliposomes, LmrA-mediated ethidium transport in metabolically active cells is dependent on both ion coupling and ATP binding and hydrolysis (Fig. 4).

Our findings indicate a fundamental link between the chemiosmotic mechanisms of secondary-active transporters (*30*) and the transport mechanism of LmrA and demonstrate the involvement of extra steps (ATP binding and hydrolysis) to retrieve and transport membrane-soluble substrates from the phospholipid bilayer. The findings highlight novel mechanistic features of substrate translocation pathways in this ABC transporter and provide a link to early hypotheses in which multidrug transporters were proposed to function as (i) aqueous pores that transport drugs from the intracellular to extracellular water phase, (ii) hydrophobic “vacuum cleaners” that transport drugs from the membrane to the extracellular environment, and (iii) lipid flippases that transport drugs from the inner to the outer leaflet of the phospholipid bilayer (*31*, *32*). In addition to small-molecule drugs, LmrA can transport a lipophilic phosphatidylethanolamine (PE) derivative (*33*) and lipid-A (*34*). LmrA shares this ability with its homolog MsbA in Gram-negative bacteria (*35*–*37*). On the basis of our observations on the LmrA-mediated transport of lipophilic ethidium, we predict that ATP binding and hydrolysis regulate the translocation of lipid substrate along its route from one membrane leaflet to the other. In the ATP-bound transporter, the ion-coupled reaction might contribute energetically to the directional movement of the hydrophilic head group of the lipid and/or to steps in the transport cycle that are not directly regulated by nucleotide.

Members of the ABC superfamily share structural motifs and features but exhibit a remarkable diversity in molecular mechanisms (*38*). ABC multidrug exporters might therefore not all operate in an identical fashion or follow a single overarching blueprint. Recent studies on proton-drug antiport by *E. coli* MsbA (*12*) and the conservation of residues E314 (*15*), N137 (fig. S6), and Q211 in ABC multidrug efflux pumps in a variety of organisms suggest that our conclusions will be relevant for other ABC exporters. However, a comparison of our findings for LmrA and MsbA suggests that the identity of ions, directions of ion transport, and ion stoichiometries might be protein-specific. Understanding how nucleotides embrace the ion-motive force in ABC transporters is important for the expansion of knowledge of the mechanisms and evolution of these transport systems. This knowledge will create new opportunities to impose therapeutic modulation on these fascinating and medically important membrane proteins. For decades, researchers have worked on deciphering the basic mechanisms by which ABC transporters operate, at present time with emphasis on the application of crystallographic and cryogenic electron microscopy techniques. Our work highlights novel dynamic features in ABC exporters that are not predicted in the static structural snapshots at the current resolution. Detailed biochemical, biophysical, and electrophysiological studies therefore remain essential for our ability to identify new principles in the mechanisms of membrane transporters.

## MATERIALS AND METHODS

### Bacterial strain and growth conditions

N-terminal hexa-histidine–tagged wild-type LmrA and its mutants—LmrA-MD, LmrA-K32E R34E (EE), LmrA-ΔK388, and LmrA-E314A—were expressed from pNZ8048-derived plasmids in the drug-hypersensitive *L. lactis* strain NZ9000 Δ*lmrA* Δ*lmrCD* using the Nisin Controlled Gene Expression (NICE) system (*10*, *33*, *39*). The LmrA-N137A mutant was generated by site-directed mutagenesis using the forward primer 5′-CTCGCTTAGCT**GCG**GATACCACGC-3′ and the reversed primer 5′-GCGTGGTATC**CGC**AGCTAAGCGAG-3′. Cells were grown at 30°C in M17 medium (Oxoid Ltd.) supplemented with 25 mM glucose and chloramphenicol (5 μg ml^−1^).

### Preparation of inside-out membrane vesicles

Inside-out membrane vesicles (ISOVs) were prepared as described previously (*33*) with modifications. Cells were grown to OD_660_ (optical density at 660 nm) of 0.6 after which protein expression was induced by the addition of nisin (1:1000). Cells were harvested by centrifugation at 13,000*g* for 10 min at 4°C. The cell pellet was washed and resuspended in 100 mM potassium HEPES (pH 7.0) at 4°C. Lysozyme (2 mg ml^−1^; Sigma-Aldrich) and complete protease inhibitor mixture (Roche Applied Science) was added, and the resultant mixture was incubated for 30 min at 30°C. Cells were broken by passage twice through a cell disruptor (Constant Systems) at 137.9 meganewton m^−2^. Subsequently, deoxyribonuclease (DNase; 10 μg ml^−1^; Sigma-Aldrich) and 10 mM MgSO_4_ were added, and the mixture was incubated for 30 min at 30°C. Potassium EDTA (pH 7.4) was then added to a final concentration of 15 mM. Unbroken cells and cell debris were removed by centrifugation at 13,000*g* for 45 min at 4°C. ISOVs were harvested by centrifugation at 125,000*g* for 1 hour at 4°C and resuspended to a protein concentration of about 40 mg ml^−1^ in 50 mM potassium HEPES (pH 7.4) containing 10% glycerol and an EDTA-free protease inhibitor cocktail (Roche). ISOVs were stored in 2 ml of aliquots in liquid nitrogen. The expression of LmrA proteins in membrane vesicles was assessed on Coomassie-stained SDS–polyacrylamide gel electrophoresis (figs. S1 and S5) and immunoblots probed with primary mouse anti-polyhistidine tag antibody (Sigma-Aldrich, catalog no. H1029) and secondary goat anti-mouse antibody (Sigma-Aldrich, catalog no. A4416) used at dilutions of 1:1000 and 1:5000, respectively.

### Purification of LmrA proteins

His_6_-tagged LmrA-WT or mutants were purified from ISOVs by affinity chromatography (*33*) with significant modifications. Membrane protein was solubilized at 4°C for 2 hours in a buffer containing 50 mM potassium HEPES (pH 8.0), 0.3 M NaCl, 10% (v/v) glycerol, 1% (w/v) *n*-decyl-β-d-maltopyranoside (DM; Affymetrix) and 0.5% (w/v) fos-choline-12 (FC-12; Affymetrix). Unsolubilized protein was removed by centrifugation at 164,000*g* for 40 min at 4°C. Solubilized membrane proteins were mixed and incubated for 2 hours at 4°C with Ni–nitrilotriacetic acid resin (10 mg of LmrA per milliliter of resin), which was pre-equilibrated in buffer A containing 50 mM potassium HEPES (pH 8.0), 0.1 M NaCl, 25 mM imidazole, 10% (v/v) glycerol, 0.2% (w/v) DM, and 0.1% (w/v) FC-12. Following incubation, the resin was transferred to a gravity-flow column (Bio-Rad) and washed with 15 resin volumes of buffer A (pH 8.0) and 15 resin volumes of buffer B [50 mM imidazole (pH 7.0)]. Bound protein was eluted with buffer C (pH 7.0) supplemented with 200 mM imidazole, and the concentration of the protein was determined using the micro bicinchoninic acid (BCA) protein assay kit (Pierce). Eluted protein was immediately used for reconstitution into giant unilamellar vesicles (GUVs) or directly used in lipid bilayer recordings. All handlings were carried out at 4°C.

### Mass spectrometry

Purified LmrA proteins were reduced (dithiothreitol) and alkylated (iodoacetamide) and subjected to enzymatic digestion with chymotrypsin overnight at 37°C. After digestion, the supernatant was pipetted into a sample vial and loaded onto an autosampler for automated LC-MS/MS analysis. All LC-MS/MS experiments were performed using a Dionex Ultimate 3000 RSLC nanoUPLC (Thermo Fisher Scientific Inc.) system and a Q Exactive Orbitrap mass spectrometer (Thermo Fisher Scientific Inc.). Separation of peptides was performed by reverse-phase chromatography at a flow rate of 300 nl min^−1^ and a Thermo Fisher Scientific reverse-phase nano EASY-Spray column (Thermo Fisher Scientific PepMap C18; particle size, 2 μm; pore size, 100 Å; inner diameter, 75 μm; length, 50 cm). Peptides were loaded onto a precolumn (Thermo Fisher Scientific PepMap C18; particle size, 5 μm; pore size, 100 Å; inner diameter, 300 μm; length, 5 mm) from the Ultimate 3000 autosampler with 0.1% formic acid for 3 min at a flow rate of 10 μl min^−1^. After this period, the column valve was switched to allow elution of peptides from the precolumn onto the analytical column. Solvent A was water + 0.1% formic acid, and solvent B was 80% acetonitrile and 20% water + 0.1% formic acid. The linear gradient used was 2 to 40% solvent B in 30 min.

The LC eluent was sprayed into the mass spectrometer by means of an EASY-Spray source (Thermo Fisher Scientific Inc.). All mass/charge ratio (*m*/*z*) values of eluting ions were measured in an Orbitrap mass analyzer set at a resolution of 70,000 and was scanned between *m*/*z* 380 and 1500. Data-dependent scans (top 20) were used to automatically isolate and generate fragment ions by higher-energy collisional dissociation (HCD; normalized collision energy, 25%) in the HCD collision cell, and measurement of the resulting fragment ions was performed in the Orbitrap analyzer set at a resolution of 17,500. Singly charged ions and ions with unassigned charge states were excluded from being selected for MS/MS, and a dynamic exclusion window of 20 s was used.

After run, the data were processed using Protein Discoverer (version 2.1, Thermo Fisher Scientific). Briefly, all MS/MS data were converted to Mascot generic format (mgf) files, and the files were then submitted to the Mascot search algorithm (Matrix Science) and searched against a customized *L. lactis* database (IL1403 DB, 2226 sequences) containing the His-tagged LmrA-WT and LmrA-ΔK388 sequences. The database also contained common contaminant sequences (115 sequences, 38,274 residues). Variable modifications of oxidation (M), deamidation (NQ), and carbamidomethyl were applied. The peptide and fragment mass tolerances were set to 5 ppm and 0.1 Da, respectively. A significance threshold value of *P* < 0.05 and a peptide cutoff score of 20 were also applied. The exponentially modified protein abundance index (tables S1 and S2) can be used as a measure for protein abundance (*40*). Protein contaminants, for which only one unique peptide sequence was identified, are considered to be nonsignificant.

### Preparation of GUVs

A lipid mixture composed of 1,2-diphytanoyl-*sn*-glycero-3-phosphocholine (8.5 mg ml^−1^; DPhPC; Avanti Polar Lipids) and cholesterol (0.37 mg ml^−1^; Sigma-Aldrich) was prepared in chloroform (Sigma-Aldrich). GUVs were prepared by the electroformation method (*41*, *42*) with the Vesicle Prep Pro equipment (Nanion). The lipid mixture (30 μl) was placed on a glass slide [coated with conductive indium tin oxide (ITO)] and allowed to dry. A rubber O-ring was placed around the dried lipid, and 500 μl of 1 M sorbitol (Sigma-Aldrich) was added to the lipid film. The second glass slide was placed on top of the ring with the ITO coating side down, thereby allowing the conduction of current. GUVs were generated with an alternating 6-V peak-to-peak voltage at a frequency of 10 Hz for 3 hours at 40°C with a rise time of 3 min and a fall time of 5 min.

To enable recording of transporter currents, freshly purified LmrA protein was incorporated into GUVs. GUVs were made in 1 M sorbitol after which the suspension was supplemented with 100 mM NaCl (*42*). Next, 0.5 to 2 pmol (for single-transporter measurements) or 5 to 20 pmol (for macroscopic measurements) of purified LmrA protein were added to this mixture in a total volume of 200 μl. Elution buffer (containing detergent) without protein was also added to additional GUV samples. The mixture was incubated at 30°C for 1 hour. Detergent was subsequently removed through incubation with Biobeads (50 mg ml^−1^; BioRad) for 1 hour at 20°C. Before use, the beads were hydrated using methanol followed by ethanol and ultrapure H_2_O and then pre-equilibrated in 1 M sorbitol and 100 mM NaCl.

### Electrophysiological recordings

Patch-clamp experiments were conducted with the Port-a-Patch setup (Nanion). The aperture of the borosilicate chip on which the phospholipid bilayer is formed mimics the electrode tip that makes contact with the cell membrane on a large-scale electrophysiology rig. To the intracellular side, 5 μl of intracellular solution (sterile and filtered) was added. To the extracellular side, 20 μl of extracellular solution (sterile and filtered) was added. Planar lipid bilayers were formed from the capture (via suction) and subsequent bursting of GUVs on the 1-μm-diameter aperture on the chip. Negative pressure (−20 to −30 mbar) was applied, and upon the formation of a gigohm seal, excess GUVs were washed out.

*I*-*V* measurements were made by holding the lipid bilayer at 0 mV and by applying a voltage step protocol that ranged from −100 to +100 mV in 10-mV increments in which the external solution was kept at zero voltage. Each sweep lasted 1 s. To examine ion transport, ion gradients were imposed. The internal solution contained 10 mM NaCl, 10 mM HEPES, 20 mM EGTA (pH 7.2, NaOH), and commensurate sucrose to balance the osmolality. The external solution was initially identical to the internal solution or was asymmetric with a NaCl concentration of 150 mM. The liquid junction potentials between the electrode and the bath solution were calculated, and the measurements were compensated as required. In ion substitution experiments, Cl^−^ salts were used to replace all Na^+^ ions with either Li^+^ or K^+^. To examine the Cl^−^ contribution to current, the internal solution was altered to 5 mM Na_2_SO_4_, and the external solution was 45 mM Na_2_SO_4_ and 5 mM NaCl [EGTA and HEPES maintained (pH 7.2)]. *I*-*V* measurements in response to the application of nucleotides were conducted using symmetrical internal and external solutions composed of 10 mM NaCl, 50 mM KCl, 10 mM HEPES, and 20 mM EGTA (pH 7.2). Mg-nucleotide (5 mM) was added to the external solution.

By convention, we refer to the movement of positive current (that is, cations moving from the internal to external solution) as outward. Because of the external addition of ATP, only LmrA protein that was inserted in the lipid bilayer in an inside-out orientation, with the NBDs facing the external compartment, was accessible to ATP binding. The inward current is therefore equal to physiological efflux in the setting of the cell.

#### Ion stoichiometry

For detection of alterations in *E*_rev_ due to ion composition, the following Nernst equation was used

$$E_{\text{rev}}=\frac{1}{z_{\text{Na}}n_{\text{Na}}-z_{\text{Cl}}n_{\text{Cl}}-z_{H}n_{H}-z_{D}n_{D}}*\frac{2.303RT}{F}\text{log}[{\left(\frac{{\left[{\text{Na}}^{+}\right]}_{\text{out}}}{{\left[{\text{Na}}^{+}\right]}_{\text{in}}}\right)}^{n_{\text{Na}}}*{\left(\frac{{\left[{\text{Cl}}^{‐}\right]}_{\text{out}}}{{\left[{\text{Cl}}^{‐}\right]}_{\text{in}}}\right)}^{-n_{\text{Cl}}}*{\left(\frac{{\left[H^{+}\right]}_{\text{out}}}{{\left[H^{+}\right]}_{\text{in}}}\right)}^{-n_{H}}*{\left(\frac{{\left[D^{+}\right]}_{\text{out}}}{{\left[D^{+}\right]}_{\text{in}}}\right)}^{-n_{D}}]$$

in which *z*_Na_, *z*_Cl_, *z*_H_, and *z*_D_ represent the charge of the ions and *n*_Na_, *n*_Cl_, *n*_H_, and *n*_D_ are the number of each of the ions moving stoichiometrically per transport cycle. *R* is the gas constant 8.314 J K^−1^ mol^−1^, *F* is the Faraday constant 96,485 C mol^−1^, and *T* is temperature in kelvin (298 K). There is a linear relationship between *E*_rev_ and the log_10_ of the external ion concentration; the slope was used to determine the *n* values (see data analysis S1). This was achieved by maintaining the internal concentration of Na^+^ and Cl^−^ at 60 mM and varying the external concentrations from 60 to 100, 150, or 200 mM using Na_2_SO_4_ (to maintain the Cl^−^ concentration) or MgCl_2_ (to maintain the Na^+^ concentration). Mg-ATP (5 mM) was added to the external solution, and all solutions were buffered with 10 mM HEPES (pH 7.2, NaOH). Sucrose was added to the internal solution to balance osmolality. To measure the effects of HEPES on *E*_rev_, solutions contained 10 mM HEPES and 10 mM NaCl (pH 6.5, NaOH). The effect of the changes in the external HEPES concentration was examined at 10, 25, 100, and 125 mM HEPES. The amount of Na^+^ on each side of the bilayer was balanced by the addition of Na_2_SO_4_ to the internal solution (SO_4_^2−^ is not transported).

#### Single-transporter currents

Steady-state single-transporter currents were recorded with the addition of 1 to 50 fmol of LmrA protein to a preformed DPhPC bilayer or with LmrA-containing GUVs. LmrA bilayers were held at −50 mV, and Mg-ATP (or the indicated nucleotide) was applied. Both the internal and external solutions were composed of 10 mM NaCl, 50 mM KCl, 10 mM HEPES, and 20 mM EGTA (pH 7.2), and 5 mM of the indicated nucleotide was added to the external solution. Only lipid bilayers with resistances greater than 8 gigohms were used. All measurements were amplified (gain = 100 mV per picoampere) and low-pass–filtered at 3 kHz (HEKA EPC10 USB; four-pole Bessel), sampled at 50 kHz, and written into digital files with Patchmaster v2x73.5 acquisition software.

#### Analysis of single-channel recordings

Analysis was completed on up to 1-min sections of response that represent as few active proteins as possible during a cluster of bursts (without stacking of signals) and that required minimal processing. Current recordings were transferred to the QUB Classic analysis package v2.0.0.20 (https://milesculabs.biology.missouri.edu/QuB_Downloads.html, University at Buffalo, Buffalo, NY); the baseline was corrected, and the data were filtered at 1 to 2 kHz. Idealization of the record was completed using the segmental K-means (*43*) algorithm with a dead time between 0.25 and 0.5 ms. Each conductance class produced mean amplitude, active probability (equivalent to conventional *open probability* in channel measurements), mean nonactive (*mean closed*) time, and mean active (*mean open*) time. The active frequency (*open frequency*) was calculated by dividing the number of events by the total time period. Terms in italics refer to conventional ion channel parameters.

### Ethidium transport in proteoliposomes

For reconstitution of LmrA proteins into liposomes, acetone and ether-washed *E. coli* total lipid extract was mixed with l-α-phosphatidylcholine (PC) at a ratio of 3:1 (w/w) according to previously described methods (*11*, *33*). Solvent was evaporated under N_2_ gas, and lipids were hydrated in buffer D [10 mM K-HEPES buffer (pH 6.8) containing 100 mM Na acetate, 100 mM KSCN, and 50 mM KCl] and sonicated calf thymus DNA (1 mg ml^−1^; Trevigen). Subsequently, the lipid mixture was extruded 11 times through a 400-nm polycarbonate filter using a 1 ml of LiposoFast-Basic extruder (Avestin Europe GmbH) to form unilamellar liposomes. These liposomes were destabilized by step-wise addition of aliquots of 10% Triton X-100, and the titration was followed at OD_540_. Protein was added to lipid at a ratio of 1:50 (w/w), and the mixture was incubated for 30 min at room temperature, allowing the unidirectional reconstitution of the purified proteins in an inside-out orientation (*11*). Detergent was removed using polystyrene bio-beads (Bio-Bead SM-2, Bio-Rad). Bio-beads were first added at 80-mg bio-beads per milliliter of liposomes, followed by incubation for 2 hours at room temperature. This was followed by two subsequent incubations with 8-mg bio-beads per milliliter of liposomes for 2 and 18 hours at 4°C. Proteoliposomes were harvested by centrifugation at 164,000*g* for 30 min, resuspended in buffer D, and incubated for 20 min with DNase (10 μg ml^−1^) and 10 mM MgSO_4_. The proteoliposomes were recentrifuged at 164,000*g* for 30 min and resuspended in 100 to 200 μl of buffer D. Samples were maintained on ice and used for the transport assays within 40 to 45 min.

Ethidium transport by LmrA proteins in response to different ion gradients and nucleotides was measured by diluting DNA-loaded proteoliposomes 100-fold to a final concentration of 20 μg of membrane protein in 2 ml of external buffers in a 3-ml fluorescence cuvette (*5*, *11*, *15*). External buffers used to impose gradients were as follows: (i) buffer E [10 mM K-HEPES (pH 6.8), 50 mM K_2_SO_4_, and 50 mM KCl] to impose a Δp_Na_ (interior positive and high) plus ΔpH (interior alkaline) and (ii) buffer F [10 mM K-HEPES (pH 6.8) and 150 mM KCl] to impose a Δp_Na_ (interior positive and high) plus ΔpH (interior alkaline) plus ΔpCl (interior low). After 20 to 30 s of recording, ethidium bromide (2 μM) was added, and fluorescence was measured as a function of time in an LS-55B luminescence spectrometer (PerkinElmer Life Sciences) with excitation and emission wavelengths of 500 and 580 nm with slit widths of 10 and 5 nm. As a transport control in the absence of ion gradients, proteoliposomes were diluted 100-fold in the buffer D in which they were prepared. To measure the effect of ATP on the transport, 5 mM Mg-ATP or 5 mM Mg–AMP-PNP was added before the addition of ethidium. Where indicated, Na_3_-orthovanadate was included at a 1 mM concentration.

### Ethidium transport in cells

In ethidium accumulation measurements, LmrA-expressing and control cells were harvested and washed three times with 50 mM KPi (pH 7.0) containing 5 mM MgSO_4_ and resuspended in the same buffer to a final OD_660_ of 5.0 (*5*). Cell suspensions were diluted 10-fold in the same buffer supplemented with 25 mM glucose to a final volume of 2 ml in a 3-ml glass cuvette. The mixture was left for 5 min at 20°C under mild stirring to allow cells to generate metabolic energy. The ethidium fluorescence was measured with excitation and emission wavelengths of 500 and 580 nm and slit widths of 5 and 10 nm, respectively, in an LS-55B Luminescence Spectrometer (PerkinElmer Life Sciences). At *t* = 30 s, 2 μM ethidium bromide was added, and ethidium fluorescence was followed for 10 min. The initial ethidium accumulation rates [arbitrary units (a.u.) per second] were determined from the slope of the linear uptake between *t* = 50 and 110 s. They are presented as a percentage of the rate in the absence of salt. In ethidium efflux measurements, cells were first incubated with 0.6 mM of the uncoupler 2,4-dinitrophenol for 40 min at 30°C to deprive the cells of metabolic energy (*15*). The cells were then washed three times and resuspended in 50 mM KPi (pH 7.0) containing 5 mM MgSO_4_ to a final OD_660_ of 5.0. The concentrated cell suspension was diluted 10-fold in the same buffer to a final volume of 2 ml in a 3-ml glass cuvette. Ethidium bromide (2 μM) was added to the mixture, and the ethidium fluorescence was monitored. At a fluorescence level between 140 and 150 a.u., Na^+^ was added where indicated, followed by 25 mM glucose to provide cells with the metabolic energy for ethidium efflux.

### Ethidium resistance

Drug-hypersensitive *L. lactis* NZ9000 Δ*lmrA* Δ*lmrCD* cells harboring pNZ8048-based plasmids for nisin-inducible expression of LmrA-WT or LmrA-ΔK388 were grown for approximately 16 hours at 30°C in M17 medium containing 25 mM glucose and chloramphenicol (5 μg ml^−1^). The cultures were diluted fivefold in a fresh complete medium with continued incubation at 30°C. Once the cell suspension had reached an OD_660_ of 0.4, a 1:1000 dilution of the culture supernatant of the nisin producer *L. lactis* NZ9700 was added, after which growth was continued for 1 hour. The cells were subsequently diluted to an OD_660_ of 0.02 in a complete medium containing nisin and with additions of NaOH and NaCl, as indicated in the legend to Fig. 4 (G and H). The diluted cells were dispensed in the wells of a 96-well plate to which 4 μM ethidium was added. Growth was followed over time at OD_660_ at 30°C in a Versamax microplate reader (Molecular Devices).

### Intracellular pH measurements

Loading of cells with 30 μM of the pH-sensitive probe CFDASE (Molecular Probes), and measurements of proton transport were performed as previously described (*10*).

### Analysis of the protein data bank

Binding sites for Na^+^ or Cl^−^ were individually identified in protein structures determined through x-ray diffraction at resolutions of 3.0 Å or better. No two proteins with a sequence identity of 90% or greater were included in this search. Binding sites featuring three or more ion-coordinating Gln or Asn, and no Glu, Asp, Arg, or Lys, were selected among all sites initially identified; other types of contact (for example, backbone, other polar side chains, and water molecules) were not explicitly specified in the search. A putative ion-coordinating contact was considered as that for which the distance to the ion is 3.0 Å or less in the case of Na^+^ and 3.5 Å or less in the case of Cl^−^.

### Statistical information

The measured values in electrophysiological experiments are reported as means ± SEM based on at least three to five independent observations. Analysis was completed with GraphPad 6.0h software using either the paired Student’s *t* test or ANOVA with a 95% confidence interval for the sample mean. Significance of data obtained with whole cells and proteoliposomes was tested by the Student’s *t* test for data in Fig. 3 (E to H) or ANOVA for data in Figs. 4 (A, B, and F) and 5 (A and B). Asterisks directly above bars in the histograms refer to comparisons with control; asterisks above lines refer to specific comparisons: *P < 0.05, **P < 0.01, ***P < 0.001, ****P < 0.0001.

## Supplementary Material

http://advances.sciencemag.org/cgi/content/full/4/9/eaas9365/DC1

## SUPPLEMENTARY MATERIALS

Supplementary material for this article is available at http://advances.sciencemag.org/cgi/content/full/4/9/eaas9365/DC1 Fig. S1. Expression and purification of LmrA proteins. Fig. S2. Peptide coverage maps from the Mascot LC-MS/MS database search results. Fig. S3. Ion binding sites in LmrA. Fig. S4. Binding sites for Na^+^ and Cl^−^ in example proteins. Fig. S5. Expression and purification of LmrA-N137A mutant protein. Fig. S6. Conservation of residue N137 in ABC multidrug transporters. Table S1. Mascot search results for mass spectrometry data for purified LmrA-WT. Table S2. Mascot search results for mass spectrometry data for purified LmrA-ΔK388. Table S3. Speciation of HEPES at pH 6.5 as a function of the HEPES concentration. Data analysis S1. Determination of *E*_rev_ values and ion stoichiometry. Data analysis S2. Comparisons of ion transport models.
