# Supplementary material for: Powering the ABC multidrug exporter LmrA: How nucleotides embrace the ion-motive force
Source: Sci Adv. 2018 Sep 19;4(9):eaas9365. doi: 10.1126/sciadv.aas9365 (PMC6155054; doi:10.1126/sciadv.aas9365)
Supplement: http://advances.sciencemag.org/cgi/content/full/4/9/eaas9365/DC1 [file supp_4_9_eaas9365__index.html]

Science Advances | Science Advances

## Supplementary Materials

**This PDF file includes:**

- Fig. S1. Expression and purification of LmrA proteins.
- Fig. S2. Peptide coverage maps from the Mascot LC-MS/MS database search results.
- Fig. S3. Ion binding sites in LmrA.
- Fig. S4. Binding sites for Na+ and Cl− in example proteins.
- Fig. S5. Expression and purification of LmrA-N137A mutant protein.
- Fig. S6. Conservation of residue N137 in ABC multidrug transporters.
- Table S1. Mascot search results for mass spectrometry data for purified LmrA-WT.
- Table S2. Mascot search results for mass spectrometry data for purified LmrA-ΔK388.
- Table S3. Speciation of HEPES at pH 6.5 as a function of the HEPES concentration.
- Data analysis S1. Determination of *E*rev values and ion stoichiometry.
- Data analysis S2. Comparisons of ion transport models.

Download PDF

**Files in this Data Supplement:**

- Adobe PDF - aas9365\_SM.pdf
