# Supplementary material for: Powering the ABC multidrug exporter LmrA: How nucleotides embrace the ion-motive force
Source: Sci Adv. 2018 Sep 19;4(9):eaas9365. doi: 10.1126/sciadv.aas9365 (PMC6155054; doi:10.1126/sciadv.aas9365)
Supplement: http://advances.sciencemag.org/cgi/content/full/4/9/eaas9365/DC1 [file aas9365_SM.pdf]

Science Advances
AAAS

advances.sciencemag.org/cgi/content/full/4/9/eaas9365/DC1

Supplementary Materials for

Powering the ABC multidrug exporter LmrA: How nucleotides embrace the ion-motive force

Kelvin Agboh, Calvin H. F. Lau, Yvonne S. K. Khoo, Himansha Singh, Sagar Raturi, Asha V. Nair, Julie Howard, Marco Chiapello, Renata Feret, Michael J. Deery, Satoshi Murakami, Hendrik W. van Veen*

*Corresponding author. Email: hwv20@cam.ac.uk

Published 19 September 2018, *Sci. Adv.* **4**, eaas9365 (2018)
DOI: 10.1126/sciadv.aas9365

This PDF file includes:

Fig. S1. Expression and purification of LmrA proteins.
Fig. S2. Peptide coverage maps from the Mascot LC-MS/MS database search results.
Fig. S3. Ion binding sites in LmrA.
Fig. S4. Binding sites for Na^+ and Cl^- in example proteins.
Fig. S5. Expression and purification of LmrA-N137A mutant protein.
Fig. S6. Conservation of residue N137 in ABC multidrug transporters.
Table S1. Mascot search results for mass spectrometry data for purified LmrA-WT.
Table S2. Mascot search results for mass spectrometry data for purified LmrA-ΔK388.
Table S3. Speciation of HEPES at pH 6.5 as a function of the HEPES concentration.
Data analysis S1. Determination of E_{rev} values and ion stoichiometry.
Data analysis S2. Comparisons of ion transport models.

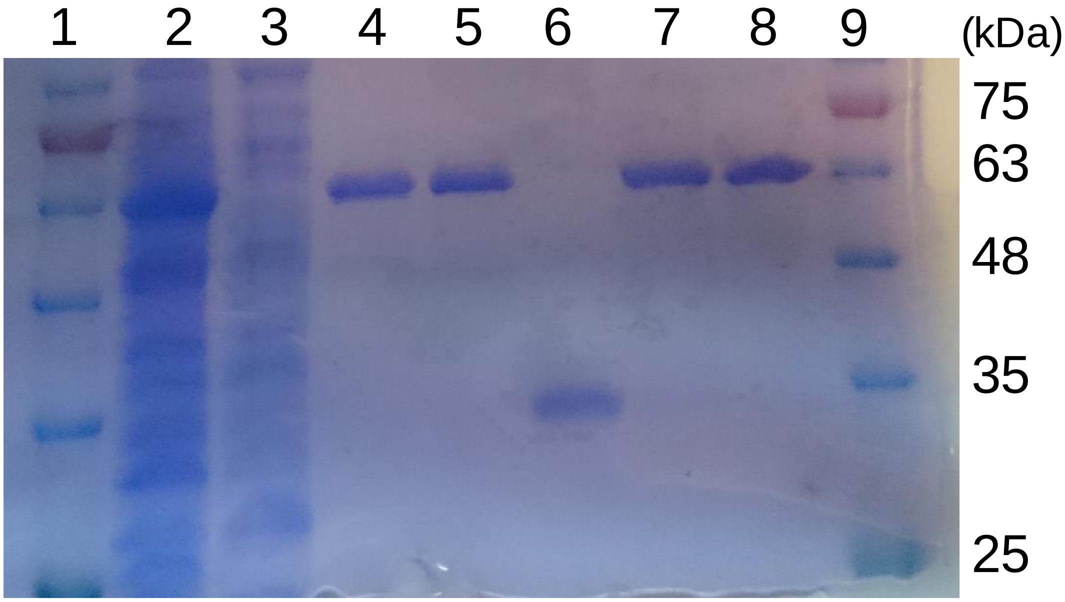

Fig. S1. Expression and purification of LmrA proteins. Proteins are shown on Coomassie-stained 10% SDS-PAGE. Total membrane proteins (20 µg per lane) in membrane vesicles prepared from cells expressing LmrA-WT (lane 2), total membrane proteins in non-expressing control cells (lane 3). Lanes 4-8 are purified protein samples (10 µg per lane) containing LmrA-WT (lane 4), LmrA-ΔK388 (lane 5), LmrA-MD protein (lane 6), LmrA-EE (lane 7), LmrA-E314A (lane 8). The migration of molecular mass markers (kDa) is indicated (lane 1 and 9).

LmrA-WT

```
  1 MGHHHHHHDD DDKAERGPQM ANRIEGKAVD KTSIKHFVKL IRAAKPRYLF
 51 FVIGIVAGII GTLIQLQVPK MVQPLINSFG HGVNGGKVAL VIALYIGSAA
101 VSAIAAIVLG IFGESVVKNL RTRVWDKMIH LPVKYFDEVK TGEMSSRLAN
151 DTTQVKNLIA NSIPQAFTSI LLLVGSIIFM LQMQWRLTLA MIIAVPIVML
201 IMFPIMTFGQ KIGWTRQDSL ANFQGIASES LSEIRLVKSS NAEKQASKKA
251 ENDVNALYKI GVKEAVFDGL MSPVMMLSMM LMIFGLLAYG IYLISTGVMS
301 LGTLLGMMMY LMNLIGVVPT VATFFTELAK ASGSTGRLTE LLDEEQEVLH
351 QGDSLDLEGK TLSAHHVDFA YDDSEQILHD ISFEAQPNSI IAFAGPSGGG
401 KSTIFSLLER FYQPTAGEIT IGGQPIDSVS LENWRSQIGF VSQDSAIMAG
451 TIRENLTYGL EGNFTDEDLW QVLDLAFARS FVENMPDQLN TEVGERGVKI
501 SGGQRQRLAI ARAFLRNPKI LMLDEATASL DSESESMVQR ALDSLMKGRT
551 TLVIAHRLST IVDADKIYFI EKGEITGSGK HNELVATHPL YAKYVSEQLT
601 VGQ
```

LmrA-ΔK388

```
  1 MGHHHHHHDD DDKAERGPQM ANRIEGKAVD KTSIKHFVKL IRAAKPRYLF
 51 FVIGIVAGII GTLIQLQVPK MVQPLINSFG HGVNGGKVAL VIALYIGSAA
101 VSAIAAIVLG IFGESVVKNL RTRVWDKMIH LPVKYFDEVK TGEMSSRLAN
151 DTTQVKNLIA NSIPQAFTSI LLLVGSIIFM LQMQWRLTLA MIIAVPIVML
201 IMFPIMTFGQ KIGWTRQDSL ANFQGIASES LSEIRLVKSS NAEKQASKKA
251 ENDVNALYKI GVKEAVFDGL MSPVMMLSMM LMIFGLLAYG IYLISTGVMS
301 LGTLLGMMMY LMNLIGVVPT VATFFTELAK ASGSTGRLTE LLDEEQEVLH
351 QGDSLDLEGK TLSAHHVDFA YDDSEQILHD ISFEAQPNSI IAFAGPSGGG
401 STIFSLLERF YQPTAGEITI GGQPIDSVSL ENWRSQIGFV SQDSAIMAGT
451 IRENLTYGLE GNFTDEDLWQ VLDLAFARSF VENMPDQLNT EVGERGVKIS
501 GGQRQRLAIA RAFLRNPKIL MLDEATASLD SESESMVQRA LDSLMKGRTT
551 LVIAHRLSTI VDADKIYFIE KGEITGSGKH NELVATHPLY AKYVSEQLTV
601 GQ
```

Fig. S2. Peptide coverage maps from the Mascot LC-MS/MS database search results. Those stretches of sequences in LmrA-WT and LmrA-ΔK388 labelled red are peptide matches. The ΔK388 difference between the two sequences is highlighted in blue.

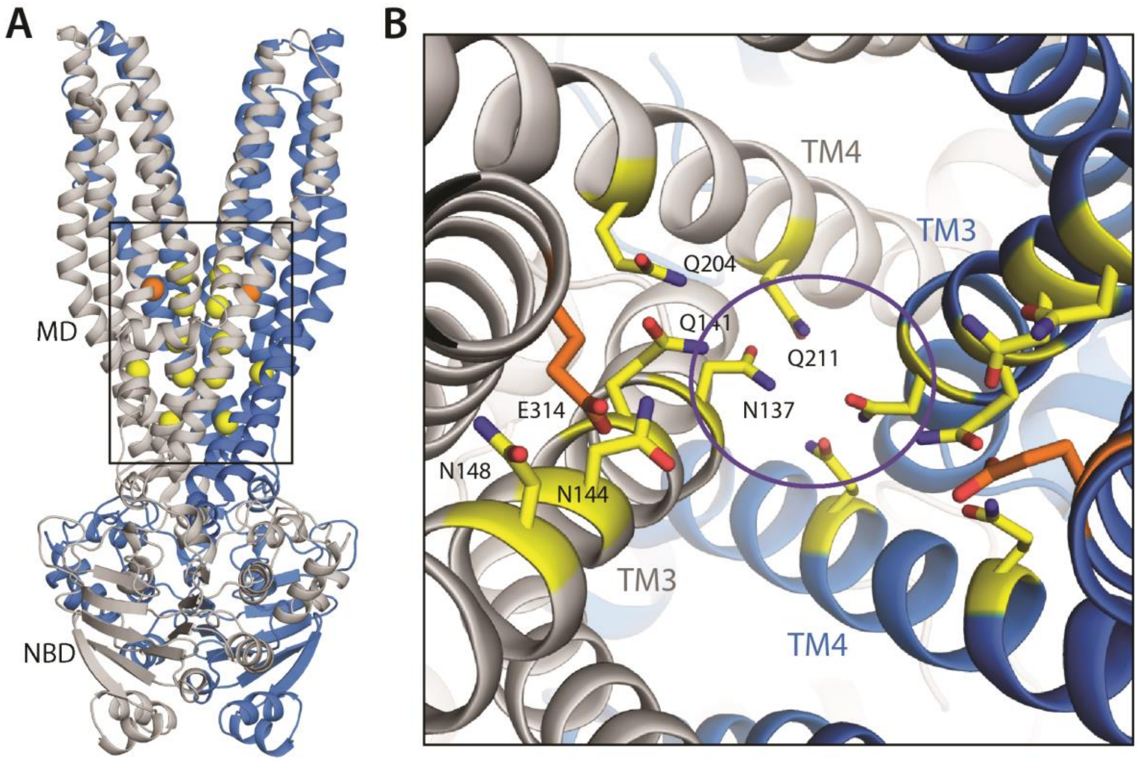

Fig. S3. Ion binding sites in LmrA. This homology model of LmrA (*16*) is based on the structure of Sav1866 in the AMP-PNP-bound outward-facing conformation (*19*). (**A**) View of the structure along the membrane plane; two identical protomers (marine, gray) form the functional complex. Each protomer consists of an intracellular nucleotide-binding domain (NBD) and a membrane domain (MD). (**B**) View of the interface between protomers from the extracellular side. Glu (orange), Gln and Asn (yellow) side-chains along helices TM3 and TM4 are highlighted. Proton-binding E314 is located in the interior cavity at the dimer interface in close proximity to an amide-containing face (Q141, N144, N148] in TM3. The bottom of the interior cavity contains 4 amide side chains, N137 (intracellular extension of TM3) and Q211 (intracellular extension of TM4) in each half-transporter (in blue circle) near the membrane-cytoplasm interface. The arrangement of these 4 amide residues is similar to those in known Na^+ and Cl^- binding sites in a variety of proteins (see fig. S4). The position of these residues along the membrane normal is indicated in (A).

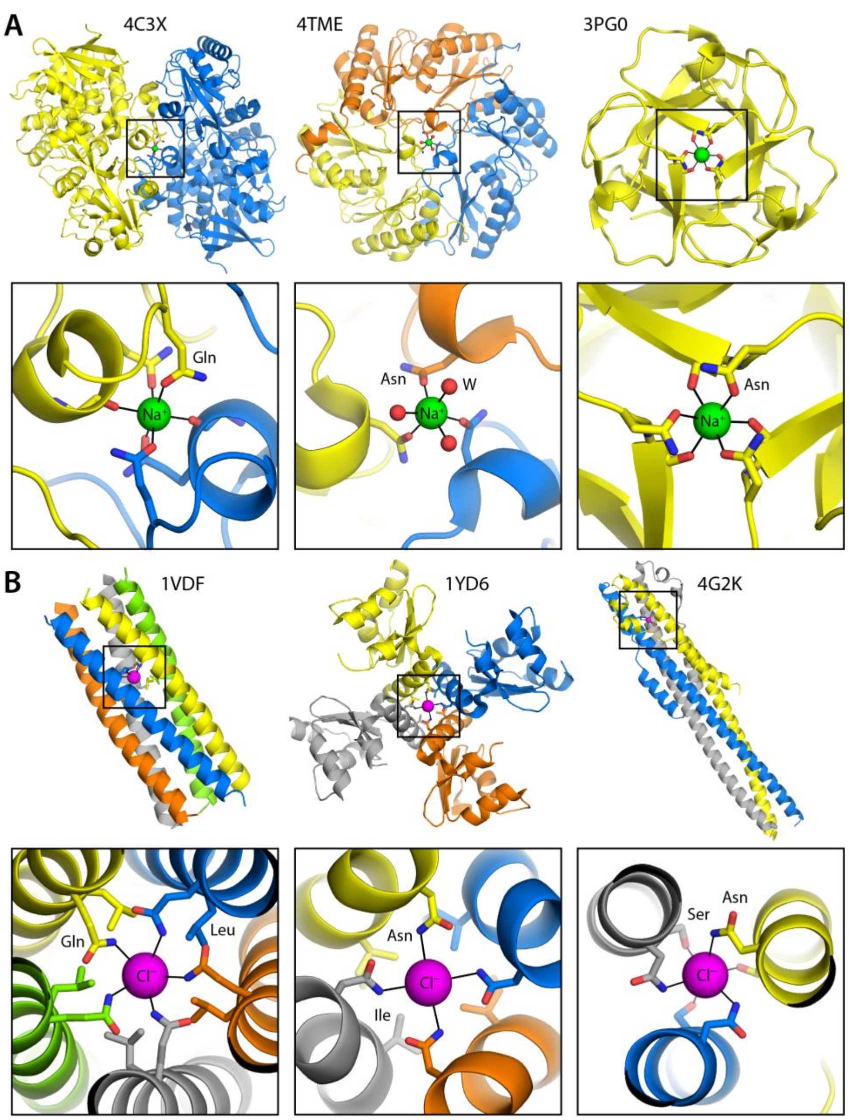

Fig. S4. Binding sites for Na^+ and Cl^- in example proteins. The structures shown are a sample of those identified in a search of the Protein Data Bank (see Material and Methods) that primarily consist of Gln or Asn residues without ionisable residues. The PDB code is specified in each case. When the structure is an oligomeric assembly, each protomer is coloured separately (yellow, marine, etc.). The groups involved in direct ion coordination (side-chain, backbone, water molecules) are highlighted. (**A**) Binding sites for Na^+, from left to right: 3-ketosteroid-delta 1-dehydrogenase from *Rhodococcus erythropolis* SQ1 (PDB 3C3X); EutL from *Clostridium perfringens* (PDB 4TME); designed β-trefoil-like protein (PDB 3PG0). (**B**) Binding sites for Cl^-, from left to right: oligomerization domain of the Cartilage Oligomeric Matrix Protein from rat (PDB 1VDF); N-terminal endonuclease domain of UvrC from *Bacillus caldotenax* (PBD 1YD6); Marburg Virus GP2 ectodomain (PDB 4G2K).

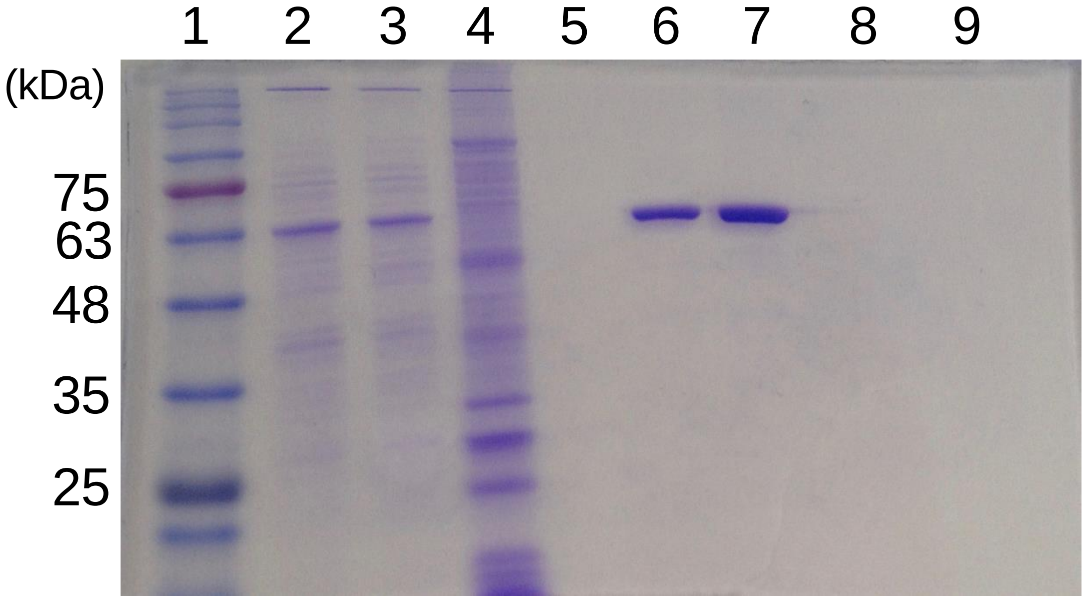

Fig. S5. Expression and purification of LmrA-N137A mutant protein. Proteins are shown on Coomassie-stained 10% SDS-PAGE. Total membrane proteins (5 µg per lane) in membrane vesicles prepared from lactococcal cells expressing LmrA-WT (lane 2) or LmrA-N137A (lane 3), or from non-expressing control cells (lane 4). Both proteins are equally well expressed in the plasma membrane. Lanes 6 and 7 are samples of purified LmrA-N137A (lane 6) and LmrA-WT (lane 7), approx. 10 µg protein per lane. The migration of molecular mass markers (kDa) is shown in lane 1.

LmrA (*Lactococcus lactis*)	129	GEMSSRLANDTTQVK	143
ABCB1-C (*human*)	812	GALTTRLANDAAQVK	826
ABCB1-N (*human*)	169	GELNTRLTDDVSKIN	183
ABCB1a-C (*mouse*)	808	GALTTRLANDAAQVK	822
ABCB1a-N (*mouse*)	165	GELNTRLTDDVSKIN	179
HorA (*Lactobacillus brevis*)	121	GEITSRLVNDSTQVK	135
Sav1866 (*Staphylococcus aureus*)	118	GQVISRVINDVEQTK	132
BmrA (*Bacillus subtilis*)	121	GETVSRVTNDTMVVK	135
MsbA (*Vibrio cholera*)	122	GGLLSRITYDSEQVA	136

Fig. S6. Conservation of residue N137 in ABC multidrug transporters. The first and last residues are numbered. ABCB1-C and ABCB1-N represent the C- and N-terminal halves of human ABCB1. ABCB1a-C and ABCB1a-N represent the C- and N-terminal halves of mouse ABCB1a.

Table S1. Mascot search results for mass spectrometry data for purified LmrA-WT.

Name		unique peptide sequences	emPAI
pNZHLmrA		39	20.59
P00766|CTRA_BOVIN	Chymotrypsinogen A OS=Bos taurus	20	200.5
P35527|K1C9_HUMAN	Keratin, type I cytoskeletal 9 OS=Homo sapiens	6	0.56
P13645|K1C10_HUMAN	Keratin, type I cytoskeletal 10 OS=Homo sapiens	6	0.6
P00761|TRYP_PIG	Trypsin OS=Sus scrofa	3	2.03
P04264|K2C1_HUMAN	Keratin, type II cytoskeletal 1 OS=Homo sapiens	3	0.23
P35908|K22E_HUMAN	Keratin, type II cytoskeletal 2 epidermal OS=Homo sapiens	3	0.23
P00767|CTRB_BOVIN	Chymotrypsinogen B OS=Bos taurus	2	0.42
P02666|CASB_BOVIN	Beta-casein OS=Bos taurus	1	0.2
Q48727|BGAL_LACLA	Beta-galactosidase OS=Lactococcus lactis subsp. lactis (strain IL1403)	1	0.04
Q9CDT9_LACLA	Competence protein ComGB OS=Lactococcus lactis subsp. lactis (strain IL1403)	1	0.12
Q9CGA1_LACLA	Uncharacterized protein OS=Lactococcus lactis subsp. lactis (strain IL1403)	1	0.52
Q9CFF6_LACLA	ABC transporter ATP binding and permease protein OS=Lactococcus lactis subsp. lactis (strain IL1403)	1	0.08
Q9CHX7_LACLA	Transcriptional regulator OS=Lactococcus lactis subsp. lactis (strain IL1403)	1	0.33
Q9CJI4_LACLA	Uncharacterized protein OS=Lactococcus lactis subsp. lactis (strain IL1403)	1	0.06
Q9CDG4_LACLA	ABC transporter ATP-binding protein OS=Lactococcus lactis subsp. lactis (strain IL1403)	1	0.2
Q9CJ47_LACLA	Uncharacterized protein OS=Lactococcus lactis subsp. lactis (strain IL1403)	1	0.08
Q9CIN1_LACLA	ABC transporter ATP binding protein OS=Lactococcus lactis subsp. lactis (strain IL1403)	1	0.08
Q9CEI9_LACLA	Uncharacterized protein OS=Lactococcus lactis subsp. lactis (strain IL1403)	1	0.23
Q9CIL5|DDL_LACLA	D-alanine--D-alanine ligase OS=Lactococcus lactis subsp. lactis (strain IL1403)	1	0.13
Q9CHZ8_LACLA	Uncharacterized protein OS=Lactococcus lactis subsp. lactis (strain IL1403)	1	0.18
Q9CED6_LACLA	Uncharacterized protein OS=Lactococcus lactis subsp. lactis	1	0.12
Q9CFX8_LACLA	Uncharacterized protein OS=Lactococcus lactis subsp. lactis	1	0.11
Q9CFF2_LACLA	Uncharacterized protein OS=Lactococcus lactis subsp. lactis	1	0.1
Q9CES5_LACLA	Glutamine ABC transporter permease and substrate binding protein protein OS=Lactococcus lactis subsp. lactis	1	0.06

Table S2. Mascot search results for mass spectrometry data for purified LmrA-ΔK388.

Chymotrypsin Digestion			
**Name**		**unique peptide sequences**	**emPAI**
pNZHLmrA-ΔK388		44	29.97
P00766|CTRA_BOVIN	Chymotrypsinogen A OS=Bos taurus	17	140.47
P35527|K1C9_HUMAN	Keratin, type I cytoskeletal 9 OS=Homo sapiens	7	0.68
P04264|K2C1_HUMAN	Keratin, type II cytoskeletal 1 OS=Homo sapiens	6	0.63
P13645|K1C10_HUMAN	Keratin, type I cytoskeletal 10 OS=Homo sapiens	4	0.37
P00761|TRYP_PIG	Trypsin OS=Sus scrofa	3	2.03
Q7DAV2_LACLA	Alpha-acetolactate synthase OS=Lactococcus lactis subsp. lactis	2	0.16
Q9CEE2|RNY_LACLA	Ribonuclease Y OS=Lactococcus lactis subsp. lactis	2	0.17
Q9CJD8_LACLA	Dihydrolipoyl dehydrogenase OS=Lactococcus lactis subsp. lactis	2	0.2
P00767|CTRB_BOVIN	Chymotrypsinogen B OS=Bos taurus	2	0.42
P02662|CASA1_BOVIN	Alpha-S1-casein OS=Bos taurus	1	0.21
P01375|TNFA_HUMAN	Tumor necrosis factor OS=Homo sapiens	1	0.2
Q9CGA1_LACLA	Uncharacterized protein OS=Lactococcus lactis subsp. lactis	1	0.52
Q9CDM5_LACLA	DNA polymerase III, subunits beta and tau OS=Lactococcus lactis subsp. lactis	1	0.08
Q48727|BGAL_LACLA	Beta-galactosidase OS=Lactococcus lactis subsp. lactis	1	0.04
P02666|CASB_BOVIN	Beta-casein OS=Bos taurus	1	0.2
Q9CJA0_LACLA	ABC transporter ATP binding protein OS=Lactococcus lactis subsp. lactis	1	0.08
Q9CG38_LACLA	ABC transporter ABC binding and permease protein OS=Lactococcus lactis subsp. lactis	1	0.07
Q9CG28|MURI_LACLA	Glutamate racemase OS=Lactococcus lactis subsp. lactis	1	0.16
Q9CHK0|MEND_LACLA	2-succinyl-5-enolpyruvyl-6-hydroxy-3-cyclohexene-1-carboxylate synthase OS=Lactococcus lactis subsp. lactis	1	0.08
Q9CJI4_LACLA	Uncharacterized protein OS=Lactococcus lactis subsp. lactis	1	0.06
Q9CHC7_LACLA	Uncharacterized protein OS=Lactococcus lactis subsp. lactis	1	0.06
Q9CGS6_LACLA	Prophage pi2 protein 11, topoisomerase OS=Lactococcus lactis subsp. lactis	1	0.21
Q9CDT9_LACLA	Competence protein ComGB OS=Lactococcus lactis subsp. lactis	1	0.12
Q9CDG5_LACLA	Uncharacterized protein OS=Lactococcus lactis subsp. lactis	1	0.13
Q01999|TRPC_LACLA	Indole-3-glycerol phosphate synthase OS=Lactococcus lactis subsp. lactis	1	0.17
P49016|MENG_LACLA	Demethylmenaquinone methyltransferase OS=Lactococcus lactis subsp. lactis	1	0.18
Q9CHV0|HPRK_LACLA	HPr kinase/phosphorylase OS=Lactococcus lactis subsp. lactis	1	0.14
Q9CFF2_LACLA	Uncharacterized protein OS=Lactococcus lactis subsp. lactis	1	0.1
Q9CEJ4|MNMG_LACLA	tRNA uridine 5-carboxymethylaminomethyl modification enzyme MnmG OS=Lactococcus lactis subsp. lactis	1	0.07
Q9CDV8_LACLA	Threonine synthase OS=Lactococcus lactis subsp. lactis	1	0.09

Data analysis S1. Determination of E_{rev} values and ion stoichiometry.

We consider a thermodynamic reaction cycle involving the coupled transport of n_{Na} sodium ions from the outside to the inside of the phospholipid bilayer, and n_{Cl} chloride ions, n_H protons, and n_D drug molecules from the inside to the outside, with z_{Na}, z_{Cl}, z_H, and z_D representing the charge of these ions. At equilibrium, the free energies of the coupled ions define a zero-flux equation relating the reversal potential (E_{rev}) to the transmembrane ion gradients:

$$E_{rev} = \frac{1}{z_{Na}n_{Na} - z_{Cl}n_{Cl} - z_H n_H - z_D n_D} * \frac{2.303\ RT}{F} \log\left[\left(\frac{[Na^+]_{out}}{[Na^+]_{in}}\right)^{n_{Na}} * \left(\frac{[Cl^-]_{out}}{[Cl^-]_{in}}\right)^{-n_{Cl}} * \left(\frac{[H^+]_{out}}{[H^+]_{in}}\right)^{-n_H} * \left(\frac{[D^+]_{out}}{[D^+]_{in}}\right)^{-n_D}\right]$$
(Eq. 1)

$$E_{rev} = \frac{1}{n_{Na} + n_{Cl} - n_H - n_D} * \frac{2.303\ RT}{F} \log\left[\left(\frac{[Na^+]_{out}}{[Na^+]_{in}}\right)^{n_{Na}} * \left(\frac{[Cl^-]_{out}}{[Cl^-]_{in}}\right)^{-n_{Cl}} * \left(\frac{[H^+]_{out}}{[H^+]_{in}}\right)^{-n_H} * \left(\frac{[D^+]_{out}}{[D^+]_{in}}\right)^{-n_D}\right]$$ **(Eq. 2)**

(A) Imposition of Na^+ gradients

With the $[Cl^-]_{in} = [Cl^-]_{out}$, $[H^+]_{in} = [H^+]_{out}$, $[HEPES^+]_{in} = [HEPES^+]_{out}$, Eq. 2 transforms into:

$$E_{rev} = \frac{1}{n_{Na} + n_{Cl} - n_H - n_D} * \frac{2.303\ RT}{F} \log\left[\left(\frac{[Na^+]_{out}}{[Na^+]_{in}}\right)^{n_{Na}}\right]$$ **(Eq. 3)**

With $[Na^+]_{in}$ = 60 mM, Eq. 3 transforms into:

$$E_{rev} = \frac{n_{Na}}{n_{Na} + n_{Cl} - n_H - n_D} * \frac{2.303\ RT}{F} \log\left[\left(\frac{[Na^+]_{out}}{60\ mM}\right)\right]$$

$$E_{rev} = \frac{n_{Na}}{n_{Na} + n_{Cl} - n_H - n_D} * \frac{2.303\ RT}{F} \log\,[Na^+]_{out} - \frac{n_{Na}}{n_{Na} + n_{Cl} - n_H - n_D} * \frac{2.303\ RT}{F} \log\ 60\ mM$$

E_{rev} was measured as a function of ${}^{10}\log\,[Na^+]_{out}$ at concentrations of 60 mM,100 mM, 150 mM and 200 mM (Fig. 2G), and followed a linear relationship $y = a \log x - b$

$$slope\ (a) = {}^{\Delta E_{rev}}\!/_{\Delta \log [Na^+]_{out}} = \frac{n_{Na}}{n_{Na} + n_{Cl} - n_H - n_D} * 59.1\ mV = 104.6 \pm 5.3\ mV\ (experimental\ value)$$

$$\frac{n_{Na}}{n_{Na} + n_{Cl} - n_H - n_D} = 1.8 \pm 0.1$$ **(Eq. 4)**

(B) Imposition of Cl^- gradients

With the $[Na^+]_{in} = [Na^+]_{out}$, $[H^+]_{in} = [H^+]_{out}$, $[HEPES^+]_{in} = [HEPES^+]_{out}$, and $[Cl^-]_{in}$ = 60 mM, Eq. 2 transforms into:

$$E_{rev} = \frac{-n_{Cl}}{n_{Na} + n_{Cl} - n_H - n_D} * \frac{2.303\ RT}{F} \log\left[\left(\frac{[Cl^-]_{out}}{60\ mM}\right)\right]$$

E_{rev} was measured as a function of $[Cl^-]_{out}$ = 60 mM, 100 mM, 150 mM and 200 mM.

In Fig. 2G: $slope = {}^{\Delta E_{rev}}/_{\Delta \log[Cl^{-}]_{out}} = -\frac{n_{Cl}}{n_{Na}+n_{Cl}-n_H-n_D} * 59.1\,mV = -59.1 \pm 7.8\,mV$ (*exp. value*)

$\frac{n_{Cl}}{n_{Na}+n_{Cl}-n_H-n_D}$ = 1.0 ± 0.1 **(Eq. 5)**

(C) Imposition of HEPES$^+$ gradients

HEPES can exist in the form of monovalent cationic, zwitterionic, and monovalent anionic species, and has two protonatable moieties, a piperazin moiety with pKa_1 of 3, and a sulfonate moiety with pKa_2 of 7.5 (20 °C) in the following equilibria:

$$H_2A^+ \overset{pKa_1}{\leftrightarrow} HA \overset{pKa_2}{\leftrightarrow} A^-$$

in which H_2A^+, HA and A^- refer to the double protonated form ($HEPES^+$, protonated on piperazin and sulfonate moiety), zwitterionic from ($HEPES^0$, protonated on piperazin moiety only) and double deprotonated form of HEPES ($HEPES^-$). Using the Henderson–Hasselbalch equation, the speciation at pH 6.5 was calculated:

Table S3. Speciation of HEPES at pH 6.5 as a function of the HEPES concentration.

HEPES	10 mM	25 mM	100 mM	125 mM
$[HEPES^+]$ (M)	$2.9*10^{-7}$	$7.3*10^{-7}$	$2.9*10^{-6}$	$3.6*10^{-6}$
$[HEPES^0]$ (M)	$9.1*10^{-4}$	$2.3*10^{-3}$	$9.1*10^{-3}$	$1.1*10^{-2}$
$[HEPES^-]$ (M)	$9.1*10^{-3}$	$2.3*10^{-2}$	$9.1*10^{-2}$	$1.1*10^{-1}$

With $[Na^+]_{in} = [Na^+]_{out}$, $[H^+]_{in} = [H^+]_{out}$ (fixed at pH = 6.5), $[Cl^-]_{in} = [Cl^-]_{out}$, $[HEPES^+]_{in}$ = $2.9*10^{-7}$ M and $[HEPES^+]_{out}$ set at $2.9*10^{-7}$ M, $7.3*10^{-7}$ M, $2.9*10^{-6}$ M or $3.6*10^{-6}$ M, Eq. 2 transforms into:

$$E_{rev} = \frac{-n_D}{n_{Na}+n_{Cl}-n_H-n_D} * \frac{2.303\,RT}{F} \log\left[\left(\frac{[HEPES^+]_{out}}{2.9 \exp -7\text{ M}}\right)\right]$$

In Fig. 2G:

$slope = {}^{\Delta E_{rev}}/_{\Delta \log[HEPES^+]_{out}} = -\frac{n_D}{n_{Na}+n_{Cl}-n_H-n_D} * 59.1\,mV = -51.1 \pm 4.9\,mV$ (*exp. value*)

$\frac{n_D}{n_{Na}+n_{Cl}-n_H-n_D}$ = 0.9 ± 0.1 **(Eq. 6)**

(D) Ion stoichiometry

Our measurements of E_{rev} yielded the following system of equations:

Eq. 4: $\frac{n_{Na}}{n_{Na}+n_{Cl}-n_H-n_D}$ = 1.8 ± 0.1, Eq. 5: $\frac{n_{Cl}}{n_{Na}+n_{Cl}-n_H-n_D}$ = 1.0 ± 0.1,

and Eq. 6: $\frac{n_D}{n_{Na}+n_{Cl}-n_H-n_D}$ = 0.9 ± 0.1

In our previous measurements of the chemical proton gradient-dependent transport of radioactive $^{36}Cl^-$ in LmrA-containing proteoliposomes, we directly established that LmrA mediates the symport of Cl^- and proton with the stoichiometry of 1:1 (*10*).

Therefore, $n_H = n_{Cl}$ **(Eq. 7)**

Our observations for symmetrical NaCl-containing buffer solutions that the ATP-induced ion conductance by LmrA-WT is directly proportional to the imposed membrane voltage (Fig. 2H) demonstrates the transbilayer movement of one positive charge per transport cycle, with the term:

$\frac{1}{n_{Na}+n_{Cl}-n_H-n_D} = 1$ in Eq. 2. Hence, $n_{Na} + n_{Cl} - n_H - n_D = 1$ **(Eq. 8)**

Taken together, Equations 4 - 8 indicate that the number of ions per transport cycle is $\boldsymbol{n_{Na} = 2}$**,** $\boldsymbol{n_{Cl} = 1}$**,** $\boldsymbol{n_H = 1}$**, and** $\boldsymbol{n_D = 1}$

In the schematic below, *in* and *out* refer to the inside and outside of the membrane in lactococcal cells.

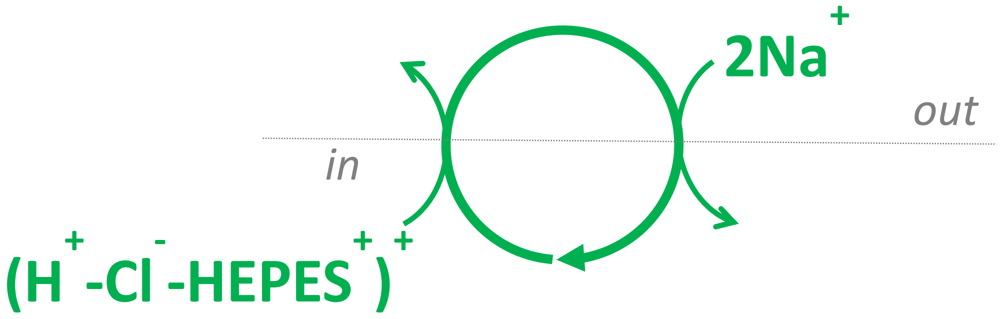

(E) Tests of the model by simultaneous changes of ion concentrations

E1. Change in $[NaCl]_{in}/[NaCl]_{out}$ from 10 mM/10 mM to $[NaCl]_{in}/[NaCl]_{out}$ 10 mM/150 mM

With $[H^+]_{in} = [H^+]_{out}$, $[HEPES^+]_{in} = [HEPES^+]_{out}$, $[Na^+]_{in} = [Cl^-]_{in} = 10$ mM, and simultaneous change in $[Na^+]_{out}$ and $[Cl^-]_{out}$ to 150 mM NaCl, Eq. 2 transforms into:

$$E_{rev} = \frac{1}{n_{Na} + n_{Cl} - n_H - n_D} * \frac{2.303RT}{F} \log \left[\left(\frac{[Na^+]_{out}}{[Na^+]_{in}}\right)^{n_{Na}} * \left(\frac{[Cl^-]_{out}}{[Cl^-]_{in}}\right)^{-n_{Cl}} \right]$$

$$E_{rev} = \frac{1}{n_{Na} + n_{Cl} - n_H - n_D} * \frac{2.303RT}{F} \log \left(\frac{150}{10}\right)^2 * \left(\frac{150}{10}\right)^{-1}$$

$$E_{rev} = \frac{1}{n_{Na} + n_{Cl} - n_H - n_D} * \frac{2.303RT}{F} \log (225 * 0.0667)$$

$$E_{rev} = \frac{1}{n_{Na}+n_{Cl}- n_H-n_D} * \frac{2.303RT}{F} \log\ 15.08$$

$$E_{rev} = \frac{1}{n_{Na}+n_{Cl}- n_H-n_D} * \frac{2.303RT}{F} * 1.176 = 69.5\ mV$$

The calculated E_{rev} value of 69.5 mV is close to the experimental E_{rev} value of 66.7 ± 6.1 mV (*n* = 3) (Fig. 2H).

E2. Change in $[NaCl]_{in}$/ $[NaCl]_{out}$ from 10 mM/10 mM to $[Na^+]_{in}/[Na^+]_{out}$ 50 mM/100 mM with $[Cl^-]_{in} = [Cl^-]_{out}$ = 50 mM

With $[H^+]_{in} = [H^+]_{out}$, $[HEPES^+]_{in} = [HEPES^+]_{out}$, $[Cl^-]_{in} = [Cl^-]_{out}$, and $[Na^+]_{in}/[Na^+]_{out}$ of 50 mM /100 mM, Eq. 2 transforms into:

$$E_{rev} = \frac{1}{n_{Na} + n_{Cl} - n_H - n_D} * \frac{2.303RT}{F} \log \left(\frac{[Na^+]_{out}}{[Na^+]_{in}}\right)^{n_{Na}}$$

$$E_{rev} = \frac{1}{n_{Na} + n_{Cl} - n_H - n_D} * \frac{2.303RT}{F} \log \left(\frac{100}{50}\right)^2$$

$$E_{rev} = \frac{1}{n_{Na} + n_{Cl} - n_H - n_D} * \frac{2.303RT}{F} \log\ 4 = 35.6\ mV$$

The calculated E_{rev} value of 35.6 mV is close to the experimental E_{rev} value of 37.6 ± 1.5 mV (*n* = 3) (Fig. 2H)

Hence, the model correctly predicts E_{rev} in response to simultaneous changes in ion gradients.

Data analysis S2. Comparisons of ion transport models.

(A) In previous work, Velamakanni and co-workers (*10*) focused on electrogenic proton-chloride symport by LmrA-MD and LmrA, and proposed that this reaction is based on apparent $(1H^+$-$1X^+$-$1Cl^-)^+$ co-transport in which X^+ can be Na^+ or $Drug^+$ (Model 1). The transport of 100 μM $^{36}Cl^-$ [added as NaCl] was measured in proteoliposomes containing transport proteins in an inside-out fashion. In the following calculations, the predicted direction of transport and accumulation of $^{36}Cl^-$ for Model 1 and $2Na^+/(1H^+$-$1Drug^+$-$1Cl^-)^+$ exchange (Model 2) are compared with the experimental data.

A1. ΔpH-dependent accumulation of $^{36}Cl^-$

To artificially impose a chemical proton gradient (ΔpH, interior alkaline), the proteoliposomes were prepared in 20 mM (K)PIPES (pH 7.6) containing 100 mM (K)acetate, and diluted 100-fold into 20 mM (K)PIPES (pH 6.8) containing 100 mM (K)MES. With the imposed $\Delta pH = 0.8$ (equivalent to -47 mV), a 4.4-fold accumulation of $^{36}Cl^-$ above the equilibration level in empty liposomes was observed (equivalent to -38 mV).

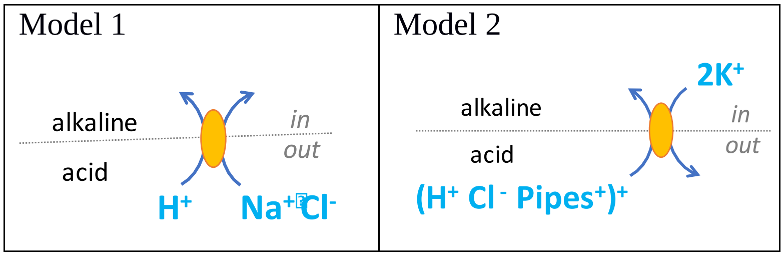

Model 1. Driving force for transport by Model 1 equals:

$$(z_{NaCl}n_{NaCl} + z_H n_H)\Delta\psi - \frac{2.303\ RT}{F}\log\left[\left(\frac{[NaCl]_{out}}{[NaCl]_{in}}\right)^{n_{NaCl}} * \left(\frac{[H^+]_{out}}{[H^+]_{in}}\right)^{n_H}\right] \quad \textbf{(Eq. 9)}$$

At steady-state with imposed ΔpH only (membrane potential $(\Delta\psi) = 0\ mV$), $z_{NaCl} = 0$, $z_H = +1$, $n_{NaCl} = 2$ and $n_H = 1$, Eq. 9 transforms into:

$$\frac{2.303\ RT}{F}\log\left[\left(\frac{[NaCl]_{out}}{[NaCl]_{in}}\right)^2 * \left(\frac{[H^+]_{out}}{[H^+]_{in}}\right)^1\right] = 0$$

$$2*59.1*\log\frac{[NaCl]_{in}}{[NaCl]_{out}} = 59.1 * \log\frac{10^{-6.8}}{10^{-7.6}}$$

$$\log\frac{[NaCl]_{in}}{[NaCl]_{out}} = \frac{59.1*\log\frac{10^{-6.8}}{10^{-7.6}}}{118.2} = 0.4$$

$$\frac{[NaCl]_{in}}{[NaCl]_{out}} = 2.5$$

With the imposed ΔpH (interior alkaline), Model 1 predicts the 2.5-fold accumulation of $^{36}Cl^-$ in the lumen of the proteoliposomes.

Model 2. As shown in Fig. 1, Na^+ in the $2Na^+/(1H^+\text{-}1D^+\text{-}1Cl^-)^+$ exchange reaction can to some extend be replaced by K^+. The driving force for transport via $2K^+/(1H^+\text{-}1D^+\text{-}1Cl^-)^+$ exchange in the presence of 100 µM $^{36}Cl^-$ would equal:

$$(z_K n_K - z_{Cl} n_{Cl} - z_H n_H - z_D n_D) * \Delta\psi - \frac{2.303\ RT}{F} \log \left[\left(\frac{[K^+]_{out}}{[K^+]_{in}}\right)^{n_K} * \left(\frac{[Cl^-]_{out}}{[Cl^-]_{in}}\right)^{-n_{Cl}} * \left(\frac{[H^+]_{out}}{[H^+]_{in}}\right)^{-n_H} * \left(\frac{[D^+]_{out}}{[D^+]_{in}}\right)^{-n_D} \right] \quad \textbf{(Eq. 10)}$$

At steady-state with $[K^+]_{in} = [K^+]_{out}$, $[D]_{in} \sim [D]_{out}$ (PIPES), $\Delta\psi = 0\ mV$, $z_{Na} = +1$, $z_{Cl} = -1$, $z_H = +1$, $z_D = +1$, $n_K = 2$, $n_{Cl} = 1$, $n_H = 1$, $n_D = 1$, and with imposed ΔpH only, Eq. 10 transforms into:

$$-\frac{2.303\ RT}{F} \log \left[\left(\frac{[Cl^-]_{out}}{[Cl^-]_{in}}\right)^{-1} * \left(\frac{[H^+]_{out}}{[H^+]_{in}}\right)^{-1} \right] = 0$$

$$\log \frac{[Cl^-]_{out}}{[Cl^-]_{in}} = \frac{-59.1 * \log \frac{10^{-6.8}}{10^{-7.6}}}{59.1} = -0.8$$

$$\frac{[Cl^-]_{in}}{[Cl^-]_{out}} = 6.3$$

Hence, the predicted ΔpH (interior alkaline)-dependent accumulation of $^{36}Cl^-$ in Model 2 of 6.3-fold is close to the experimentally observed accumulation, and is in a similar range as the predicted value in Model 1.

A2. $\Delta\psi$-dependent accumulation of $^{36}Cl^-$

To artificially impose a $\Delta\psi$, the proteoliposomes were prepared in 20 mM (K)Pipes (pH 7.6) containing 100 mM K-acetate, and diluted 100-fold into 20 mM N-methyl-D-glucosamine (NMG) Pipes (pH 7.6) containing 100 mM (NMG) acetate in the presence of valinomycin to induce a potassium diffusion potential ($\Delta\psi$, interior negative) of -118.2 mV. The proteoliposomes accumulated $^{36}Cl^-$ about 17.4-fold above the equilibration level obtained for empty liposomes.

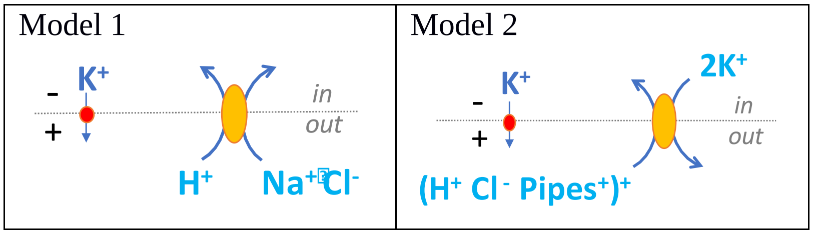

Model 1. Driving force for transport by Model 1 equals:

$$(z_{NaCl} n_{NaCl} + z_H n_H)\Delta\psi - \frac{2.303\ RT}{F} \log \left[\left(\frac{[NaCl]_{out}}{[NaCl]_{in}}\right)^{n_{NaCl}} * \left(\frac{[H^+]_{out}}{[H^+]_{in}}\right)^{n_H} \right] \quad \text{(see Eq. 9)}$$

At steady-state with $[H^+]_{in} = [H^+]_{out}$, $\Delta\psi = -118.2\ mV$, the equation transforms into:

$$\Delta\psi - 59.1 * \log\left(\frac{[NaCl]_{out}}{[NaCl]_{in}}\right)^2 = 0$$

$$-118.2 = 118.2 * \log\left(\frac{[NaCl]_{out}}{[NaCl]_{in}}\right)$$

$$\log\left(\frac{[NaCl]_{out}}{[NaCl]_{in}}\right) = -1$$

$$\frac{[NaCl]_{in}}{[NaCl]_{out}} = 10$$

With the imposed $\Delta\psi$ (interior negative), Model 1 predicts a 10-fold accumulation of $^{36}Cl^-$ in the lumen of the proteoliposomes.

Model 2. The driving force for transport by Model 2 equals:

$$(z_K n_K - z_{Cl} n_{Cl} - z_H n_H - z_D n_D) * \Delta\psi - \frac{2.303\ RT}{F} \log\left[\left(\frac{[K^+]_{out}}{[K^+]_{in}}\right)^{n_K} * \left(\frac{[Cl^-]_{out}}{[Cl^-]_{in}}\right)^{-n_{Cl}} * \left(\frac{[H^+]_{out}}{[H^+]_{in}}\right)^{-n_H} * \left(\frac{[D^+]_{out}}{[D^+]_{in}}\right)^{-n_D}\right] \text{ (see Eq. 10)}$$

At steady-state with the $[H^+]_{in} = [H^+]_{out}$, $[D^+]_{in} = [D^+]_{out}$ (PIPES), the equation transforms into:

$$\Delta\psi - \frac{2.303\ RT}{F} \log\left[\left(\frac{[K^+]_{out}}{[K^+]_{in}}\right)^{n_K} * \left(\frac{[Cl^-]_{out}}{[Cl^-]_{in}}\right)^{-n_{Cl}}\right] = 0$$

$$\Delta\psi - \frac{2.303\ RT}{F} \log\left[\left(\frac{[K^+]_{out}}{[K^+]_{in}}\right)^{n_K}\right] = \frac{2.303\ RT}{F} \log\left[\left(\frac{[Cl^-]_{out}}{[Cl^-]_{in}}\right)^{-n_{Cl}}\right]$$

with $n_K = 2$, $n_{Cl} = 1$ and $\Delta\psi = -118.2\ mV$,

$$-118.2\ mV - 59.1 * \log\left(\frac{1.2\ mM}{120\ mM}\right)^2 = -59.1 * \log\left(\frac{[Cl^-]_{out}}{[Cl^-]_{in}}\right)$$

$$\log\left(\frac{[Cl^-]_{out}}{[Cl^-]_{in}}\right) = \frac{-118.2\ mV - (59.1 * -4\ mV)}{-59.1 mV} = -2$$

$$\frac{[Cl^-]_{in}}{[Cl^-]_{out}} = 100$$

Model 2 predicts a 100-fold accumulation of $^{36}Cl^-$ in the lumen of the proteoliposomes.

Conclusions

With the imposed electrochemical ion gradients in the proteoliposomes, Model 2 and Model 1 predict Cl^- accumulation in a similar range as the experimental values obtained in the study by Velamakanni and co-workers (*10*). This conclusion is further supported by the notion that the

imposed $\Delta\psi$ (based on a potassium diffusion potential) will have been short-lived due to the export of K^+ from the lumen of the proteoliposomes via valinomycin (Model 1) and valinomycin and LmrA (Model 2).

Model 1 [$(1H^+$-$1X^+$-$1Cl^-)^+$ co-transport] was the simplest model that explained the $^{36}Cl^-$ accumulation data with the proteoliposomes. Based on our electrophysiological data and further observations in proteoliposomes and intact cells, Model 1 has now been extended to Model 2 [$(1H^+$-$1drug^+$-$1Cl^-)^+$ / $2Na^+$ antiport].

(B) Further comparisons of ion transport models

In the electrophysiological experiments in Fig. 2H, the imposition of asymmetric solutions ($[NaCl]_{in}$ /$[NaCl]_{out}$ = 10 mM/150 mM) yielded a measured E_{rev} = 66.7 ± 6.1 mV and calculated E_{rev} = 70.5 mV based on Model 2 (data analyses S1 E1).

In the following section the E_{rev} is calculated using Model 1.

$$E_{rev} = \frac{1}{(z_{NaCl}n_{NaCl} + z_H n_H)} * \frac{2.303\ RT}{F} \log \left[\left(\frac{[NaCl]_{out}}{[NaCl]_{in}} \right)^{n_{NaCl}} * \left(\frac{[H^+]_{out}}{[H^+]_{in}} \right)^{n_H} \right] \text{ (based on Eq. 9)}$$

With separate additions of Na^+ and Cl^- in the experiments in Fig. 2H, this equation can be adapted to:

$$E_{rev} = \frac{1}{n_{Na} - n_{Cl} + n_H} * \frac{2.303\ RT}{F} \log \left[\left(\frac{[Na^+]_{out}}{[Na^+]_{in}} \right)^{n_{Na}} * \left(\frac{[Cl^-]_{out}}{[Cl^-]_{in}} \right)^{n_{Cl}} * \left(\frac{[H^+]_{out}}{[H^+]_{in}} \right)^{n_H} \right]$$

At steady-state with $[H^+]_{in} = [H^+]_{out}$, $n_{Na} = 1$, $n_{Cl} = 1$ and $n_H = 1$

$$E_{rev} = \frac{2.303\ RT}{F} \log \left[\left(\frac{[Na^+]_{out}}{[Na^+]_{in}} \right)^1 * \left(\frac{[Cl^-]_{out}}{[Cl^-]_{in}} \right)^1 \right]$$

$$E_{rev} = 59.1 * \log \left[\left(\frac{150\ mM}{10\ mM} \right) * \left(\frac{150\ mM}{10\ mM} \right) \right] = 139 \text{ mV}$$

Conclusion

Model 2 has a better predictive value of E_{rev} than Model 1.
